# Aldehyde dehydrogenase 1A1 confers erlotinib resistance via facilitating the reactive oxygen species-reactive carbonyl species metabolic pathway in lung adenocarcinomas

**DOI:** 10.7150/thno.35729

**Published:** 2019-09-23

**Authors:** Hui-Min Lei, Ke-Ren Zhang, Cong Hui Wang, Yang Wang, Guang-Lei Zhuang, Li-Ming Lu, Jian Zhang, Ying Shen, Hong-Zhuan Chen, Liang Zhu

**Affiliations:** 1 Department of Pharmacology and Chemical Biology, Shanghai Jiao Tong University School of Medicine, Shanghai, 200025, China.; 2 Shanghai Collaborative Innovation Center for Translational Medicine, Shanghai, 200025, China; 3 State Key Laboratory of Oncogenes and Related Genes, Ren Ji Hospital, School of Medicine, Shanghai Jiao Tong University, Shanghai, 200127, China.; 4 Central laboratory, Shanghai Chest Hospital, Shanghai Jiao Tong University, Shanghai, 200030, China; Shanghai Institute of Immunology, Shanghai Jiao Tong University School of Medicine, Shanghai, 200025, China.; 5 Department of Pathophysiology, Key Laboratory of Cell Differentiation and Apoptosis of National Ministry of Education, Shanghai Key Laboratory of Tumor Microenvironment and Inflammation, Shanghai Jiao-Tong University School of Medicine, Shanghai 200025, China.

**Keywords:** ALDH, erlotinib resistance, reactive oxygen species, reactive carbonyl species, lung cancer.

## Abstract

**Background:** Acquired resistance to epidermal growth factor receptor (EGFR)-tyrosine kinase inhibitors (TKIs) such as erlotinib is a major challenge to achieve an overall clinical benefit of the targeted therapy. Recently, aldehyde dehydrogenase 1 (ALDH1) induction has been found to render lung adenocarcinomas resistant to EGFR-TKIs, and targeting ALDH1A1 becomes a novel strategy to overcome resistance. However, the molecular mechanism underlying such effect remains poorly understood. **Methods:** Comprehensive assays were performed in a panel of lung adenocarcinoma cell lines and xenografts that acquired resistance to erlotinib. Cancer phenotype was evaluated by cell viability, apoptosis, migration, and epithelial-mesenchymal transition analysis *in vitro*, tumorsphere formation analysis *ex vivo*, and tumor growth and dissemination analysis *in vivo*. Reactive oxygen species (ROS) and reactive carbonyl species (RCS) were detected based on fluorescent oxidation indicator and liquid chromatography coupled to mass spectrometry, respectively. Protein target was suppressed by RNA interference and pharmacological inhibition or ecto-overexpressed by lentivirus-based cloning. Gene promoter activity was measured by dual-luciferase reporting assay. **Results:** Knockdown or pharmacological inhibition of ALDH1A1 overcame erlotinib resistance *in vitro* and *in vivo*. ALDH1A1 overexpression was sufficient to induce erlotinib resistance. Metabolomic analysis demonstrated lower ROS-RCS levels in ALDH1A1-addicted, erlotinib-resistant cells; in line with this, key enzymes for metabolizing ROS and RCS, SOD2 and GPX4, respectively, were upregulated in these cells. Knockdown of SOD2 or GPX4 re-sensitized the resistant cells to erlotinib and the effect was abrogated by ROS-RCS scavenging and mimicked by ROS-RCS induction. The ALDH1A1 overexpressed cells, though resisted erlotinib, were more sensitive to SOD2 or GPX4 knockdown. The ALDH1A1 effect on erlotinib resistance was abrogated by ROS-RCS induction and mimicked by ROS-RCS scavenging. Detection of GPX4 and SOD2 expression and analysis of promoter activities of GPX4 and SOD2 under the condition of suppression or overexpression of ALDH1A1 demonstrated that the RCS-ROS-metabolic pathway was controlled by the ALDH1A1-GPX4-SOD2 axis. The ROS-RCS metabolic dependence mechanism in ALDH1A1-induced resistance was confirmed *in vivo*. Analysis of public databases showed that in patients undergoing chemotherapy, those with high co-expression of ALDH1A1, GPX4, and SOD2 had a lower probability of survival. **Conclusions:** ALDH1A1 confers erlotinib resistance by facilitating the ROS-RCS metabolic pathway. ALDH1A1-induced upregulation of SOD2 and GPX4, as well as ALDH1A1 itself, mitigated erlotinib-induced oxidative and carbonyl stress, and imparted the TKI resistance. The elucidation of previously unrecognized metabolic mechanism underlying erlotinib resistance provides new insight into the biology of molecular targeted therapies and help to design improved pharmacological strategies to overcome the drug resistance.

## Introduction

Lung cancer is the leading cause of tumor-related mortality worldwide, with adenocarcinomas representing the main histological type. Lung adenocarcinomas driven and maintained by mutant activating epidermal growth factor receptor (EGFR) respond remarkably to EGFR-tyrosine kinase inhibitors (TKIs) such as erlotinib. Despite showing promising initial responses, tumors gradually lose sensitivity to EGFR-TKIs after a period of drug administration. This ultimately happened resistance limits therapeutic efficacy and is a major challenge in treating lung adenocarcinomas. Several mechanisms contribute to the acquired resistance, such as the secondary T790M mutation in EGFR, epithelial-mesenchymal transition (EMT), and bypass pathway activation 1. In addition, other mechanisms remain to be explored. Discovering new mechanisms is essential for developing improved therapeutics to overcome resistance. Recently, aldehyde dehydrogenase 1 (ALDH1) induction has been found to render lung adenocarcinomas resistant to EGFR-TKIs, and targeting ALDH1 has emerged as a novel strategy for treating lung cancer 2-4.

How EGFR-TKIs upregulate ALDH1 has been reported. Drug stress activates β-catenin or Sox2, leading to an increase of ALDH1A1 transcription and enriching cancer stem-like cells; Notch activation by erlotinib also enriches ALDH^+^ stem-like cells 5-7. However, other than the explanation that drug resistance is attributable to its cancer stem cell (CSC) phenotype-related properties, the molecular mechanism whereby ALDH1 itself induces resistance to EGFR-TKIs remains unknown 7-9.

In the past, ALDH was considered to confer resistance to cyclophosphamide and its analogs by metabolizing and detoxifying these agents 10; however TKIs, including erlotinib, are not ALDH substrates. There must be other mechanisms whereby ALDH confers TKI resistance, rather than acting as a chemotherapeutic drug-metabolizing enzyme.

The maintenance of lower levels of reactive oxygen species (ROS) guarantees maintenance of the cancer stem state 11-13 and tumor cells can achieve anticancer drug resistance by upregulating antioxidant components or enhancing ROS metabolism 14, 15. Reactive carbonyl species (RCS), particularly reactive aldehydes derived from lipid peroxidation, are initiated by and reciprocally amplify ROS, and are prone to be metabolized by ALDH 16-19.

Herein, we find that erlotinib-resistant lung adenocarcinoma cells depend on ALDH1A1 and that ALDH1A1 confers resistance via facilitating the ROS-RCS metabolic pathway. ALDH1A1-induced upregulation of SOD2 and GPX4, as well as ALDH1A1 itself, mitigates erlotinib-induced oxidative and carbonyl stress in cancer cells and imparts erlotinib resistance. The elucidation of previously unrecognized metabolic mechanism underlying erlotinib resistance provides new insight into the biology of molecular targeted therapies and help to design improved pharmacological strategies to overcome the drug resistance.

## Materials and methods

### Cells and cell culture

Human lung adenocarcinoma cell lines HCC827 (CRL-2868) and PC9 that harbor EGFR-activating mutation were obtained from ATCC and Dr. G.L. Zhuang (China State Key Laboratory of Oncogenes and Related Genes). Cells were identified by short tandem repeat (STR) profiling. The isogenic erlotinib-resistant variants were established, maintained, and authenticated as previously described 20-22. Briefly, the parental cells were cultured in medium containing escalating concentrations of erlotinib. After 6 months of passages, the surviving cells that could grow in micromolar concentrations of erlotinib were considered resistant cells. Cells were cultured in RPMI 1640 medium (Invitrogen, USA) containing 10% FBS, 1% GlutaMAX, and 1% penicillin- streptomycin at 37 °C with 5% CO_2_.

### Reagents and cell viability assay

Erlotinib, disulfiram (DSF, Sigma-Aldrich, Germany), and N, N-diethylaminobenzaldehyde (DEAB, Sigma-Aldrich, Germany) were dissolved in Dimethyl sulfoxide (DMSO, Sigma-Aldrich, Germany) at a stock concentration of 10 mM. N-acetyl-L-cysteine (NAC, Beyotime, China) was reconstituted in PBS at a stock concentration of 1 M and adjusted pH to 7.2 with NaOH powder. Doxycycline (DOX; Aladdin, China) was dissolved in sterile water at concentration of 10 mg/mL and filtered with 0.22 μm membrane.

Cells were plated at a density of 4,000 cells/well in 96 well plates. Twenty-four hours later, drugs, siRNAs or vehicle control were added to the medium to achieve the indicated concentrations and treated for 72 h. Cell viability assays were performed by Cell Counting Kit-8 (Dojindo, Japan) according to the manufacturer's instructions. The optical density (OD) of each well was detected at 450 nm using a microplate reader (Scientific Vario, Thermo Scientific, USA). The inhibition of cell viability was calculated as [1 - (ODtreated - ODblank) / (ODvehicle control - ODblank)] × 100%.

### Cell apoptosis assay

Apoptotic and viable cells were detected using an Annexin V/PI apoptosis detection kit (BD Pharmingen, USA) according to the manufacturer's instructions. A total of 10,000 cells were analyzed per sample by a flow cytometer (Accuri C6, BD Bioscience, USA).

### Cell migration assay

The cell migration assay was performed using transwell chambers (Costar, USA). The cells were suspended in serum-free medium at a density of 50,000 cells/well and placed in the upper chamber. The lower chamber was filled with medium supplemented with 10% FBS. After an incubation period of 20 h at 37 °C with 5% CO_2_, the cells on the upper surface of the membrane were removed with a cotton-tipped swab. Cells adhering to the bottom surface of each membrane were fixed with 4% paraformaldehyde in PBS, then stained with 0.1% crystal violet solution and imaged (Nikon, Japan). Cell migration ability was quantified by dissolving stained cells in 10% acetic acid and detecting the optical density (OD) at 600 nm using a microplate reader.

### Tumorsphere formation assay

The cells were suspended in StemXVivo Serum-Free Tumorsphere Media (R&D, USA), supplemented with 2 U/mL heparin (Tocris, USA) and hydrocortisone (Tocris, USA), then seeded at 3×10^4^/well in 6-well ultralow adhesion culture plates (Costar, USA) and cultured at 37 °C with 5% CO_2_ for 7 days to form tumorspheres.

### Western blot analysis

Proteins were extracted as follows: Briefly, cells were washed with PBS, lysed in radio-immunoprecipitation assay (RIPA) buffer containing 1 mM PMSF (Beyotime, China) and placed on ice for 30 min, then centrifuged at 13,800 × *g* for 10 min. Then, the supernatant was collected and the protein concentration was determined via BCA Protein Assay Kit (Thermo Fisher, USA).

Proteins were separated by 10% SDS-PAGE gel and electro-transferred to polyvinylidene difluoride (Millipore, USA). Membranes were blocked with 5% nonfat milk in 1 × TBST and 0.1% Tween 20 for 1 h at room temperature, incubated with diluted primary antibodies in 5% BSA, 1 × TBS, and 0.1% Tween 20 at 4 °C with gentle shaking overnight. β-actin was used as an internal loading control. After incubation with corresponding secondary antibodies (CST, USA), the membranes were incubated with ECL substrate (Thermo Fisher, USA) and scanned using an imaging system (Odyssey FC, LI-COR Biosciences, USA).

### Immunofluorescence analysis

Cells were seeded in glass bottom culture dishes and cultured for 24 h. After fixation with 4% paraformaldehyde in PBS for 30 min, the cells were permeated with 0.4% triton in PBS for 20 min, blocked with 3% BSA for 1 h, and incubated with primary antibodies containing CD44 (1:500, CST, USA), E-cadherin (1:500, CST, USA), or vimentin (1:500, CST, USA) overnight at 4 °C. Subsequently, cells were incubated with the corresponding secondary antibodies including Alexa Fluor 555 labeled donkey anti-rabbit and Alexa Fluor 488 labeled goat anti-mouse for 1 h, stained with DAPI in PBS for 15 min and then subjected to laser confocal microscope (Leica SP8, Germany) analysis.

### ALDEFLUOR assay and ALDH^+^ cell sorting

The assay was performed using ALDEFLUOR kits (Stem Cell Technologies Inc., Canada) according to the manufacturer's instructions. Briefly, BODIPY‑aminoacetaldehyde is a non‑toxic fluorescent ALDH substrate able to freely diffuse into intact and viable cells. It is degraded by ALDH into BODIPY‑aminoacetate which is fluorescent and remains inside the cells. The fluorescence intensity is proportional to the ALDH activity when DEAB, the ALDH1 inhibitor is used to control background fluorescence. The fluorescence intensity was examined by a flow cytometer (Accuri C6, BD Biosciences, USA). For ALDH^+^ cell sorting, a FACSAria III flow cytometer (BD Biosciences, USA) was used.

### ROS detection

Intracellular ROS levels were quantified by using fluorescent oxidation indicator 2,7-dichlorodi-hydrofluorescein diacetate (DCFH-DA)-based flow cytometry or *in situ* imaging analysis according to the manufacturer's instructions (Beyotime, China). Briefly, the cells were collected at density of 1×10^6^, incubated with 10 μM DCFH-DA for 20 min at 37 °C, and washed with serum- free medium. The fluorescence intensity was examined by an Accuri C6 flow cytometer or an IncuCyte Live Cell Analysis System (Essen BioScience, USA).

Mitochondrially generated ROS were determined using a MitoSOX Red Mitochondrial Superoxide Indicator (Thermo Fisher, USA) according to the manufacturer's instructions. Briefly, cells were plated at a density of 2 × 10^5^ cells/well. Cells were collected and incubated with 5 μM MicroSOX Red in HBSS/Ca^2+^/Mg^2+^ at 37 °C for 30 min in the dark, then gently washed with PBS. The fluorescence intensity was examined at an excitation wavelength of 510 nm and an emission wavelength of 580 nm by Accuri C6 flow cytometry (Becton Dickinson, USA).

### RCS determination

The reactive carbonyl species were determined using liquid chromatography coupled to triple-quadrupole tandem mass spectrometry (LC-QqQ-MS/MS) analysis. The RCS in cell lysate were derivatized using dinitrophenylhydrazone. The analysis was performed by ultra-performance liquid chromatography (UPLC, SCIEX ExionLC, USA)-coupled to a triple quadrupole tandem mass spectrometer (SCIEX Triple Quad 4000, USA). The separation of metabolites was carried out on an Agilent ZORBAX SB-C18 column (2.1 mm × 50 mm, 5 μm, USA). The mobile phase consisted of solvent A (1 mm ammonium acetate in water) and solvent B (1 mm ammonium acetate in acetonitrile). A flow rate of 0.3 mL/min was used with a gradient elution of 70% A at 1 min, 70%-0% A for 1-9 min, maintained for 2 min at 100% B, decreased to 30% B from 12-12.2 min, and a re-equilibrated to the initial solvent from 12.2 to 15 min. The metabolites were ionized using the electrospray ionization interface operating in negative ion mode. IonSpray voltage was set at -4500 V, curtain gas was kept at 35 psi, ion source temperature was 550 °C, nebulizing gas and drying gas were 55 psi. Selective/multiple reaction monitoring (SRM/MRM) mode was used to collect mass spectral data of precursor and product ion transitions. Different fragmentor voltages were used for each metabolite. The collision energies were also optimized with respect to individual analytes between 10 and 30 eV to maximize the analyte response. Data was acquired and processed using MultiQuant software version 3.0.1 (SCIEX, USA).

### RNA extraction and real-time PCR

Total RNA from cells was extracted using an RNA extraction kit (Takara, Japan) according to the manufacturer's instructions. Reverse transcription was carried out using a RevertAid First Strand cDNA Synthesis Kit (Qiagen, Germany). Real-time PCR was performed using gene specific primers (Table S1) with SYBR Premix Ex Taq (Takara, Japan) using a LightCycler 480 II system (Roche). β-actin used as an internal control. Relative quantification was performed by the ΔΔCT method.

### RNA interference

Endogenous ALDH1A1, GPX4, or SOD2 were silenced using siRNAs (GenePharma, China) according to the manufacturer's instructions. Scrambled siRNAs were used as controls. Cells were seeded in 6-well plates at density of 2 × 10^5^/well. Twenty-four hours later, the cells were transfected with siRNAs using lipofectamine 3000 (Invitrogen). Silencing efficiency was detected using real-time PCR and western blotting. The sense sequences of siRNAs were shown in Supplementary Table S1.

### ALDH1A1 ecto-expression

Lentiviral ALDH1A1 expression or doxycycline induction-based ALDH1A1 expression clones were packaged with pHelper 1.0 and pHelper 2.0 in 293T cells. Empty vectors were used as the negative control. Lentiviral titer was detected by real-time qPCR performed in 293T cells. For transfection, HCC827 or PC9 cells were seeded in 6-well plates at density of 2 × 10^5^/well. Twenty-four hours later, the lentiviral particles were diluted with serum-free medium containing 6 μg/mL polybrene (Thermo Fisher, USA) and transfected to the cells. Positive clones were selected using 1 μg/mL puromycin after 72 h of transfection. The overexpression of ALDH1A1 was confirmed by real-time PCR and western blotting.

### Gene promoter dual-luciferase reporting assay

Cells were plated onto each well at density of 5 × 10^4^ in 24-well plates and cultured for 24 h. The cells were then co-transfected with 2 μg firefly luciferase reporters carrying GPX4 or SOD2 promotors together with 2 μg pGL4.74 plasmid encoding Renilla luciferase (Promega, USA) and 20 nM siALDH1A1 or siGPX4. Cell extracts were prepared 48 h after transfection, and luciferase activity was determined by the Dual-Luciferase Reporter Assay system according to manufacturer's instructions (Promega, USA).

### Analysis of combination effect

Treatment combinations can lead to additive, synergistic, antagonistic, or potentiative effects. A combination Index (CI) was used to evaluate the effects according to Chou-Talalay method^23^. Data were analyzed using CompuSyn software (CompuSyn Inc., USA).

### Animal study approval and *in vivo* xenografts assay

Procedures and experiments involving animal studies were approved by the Institutional Animal Care and Use Committee (IACUC) and carried out in accordance with the Animal Care and Use Rules of Shanghai Jiao Tong University School of Medicine.

A total of 3×10^6^ cells suspended in 100 μL PBS were subcutaneously inoculated into the left and right flanks of 5-week-old BALB/c nu/nu athymic mice. Subcutaneous local tumors were measured on length (L) and width (L) by a Vernier caliper every 2-3 days. The local growth rates of the tumors were analyzed by calculating the volume with the formula LW^2^/2. When tumor volume reached 300 mm^3^, the mice were randomly allocated to various treatment groups. For the drug treatment experiment, mice were orally gavaged with the indicated dosage of erlotinib and DSF (60 mg/kg/day) in 0.4% Tween 80, 0.5% methylcellulose (w/v), and sterile water unless otherwise indicated. Vehicle supplementation (0.4% Tween 80, 0.5% methylcellulose in sterile water) was used in the control mice. BSO (450 mg/kg, per 2 days) in sterile saline was intraperitoneally injected into mice when needed. For inducible overexpressed experiments, mice were treated with doxycycline hyclate (DOX) (2 mg/ml) in drinking water with 25 mg/mL sucrose after cancer cell injection. Mice were euthanized when the animal experiments reached the end or the tumor volume of the vehicle control group reached ~1500 mm^3^. After the euthanasia, tumors were picked and weighted. Detailed procedures for analysis of the metastasis of the subcutaneously inoculated tumors are described in the Supplementary Methods.

### Public clinical database analysis

Kaplan-Meier analysis of the association between the probability of overall survival (OS) of lung adenocarcinoma patients who were received chemotherapy and studied gene expression profiles. The analysis was performed by using the online KM-plotter tool (http://kmplot.com/analysis/index.php?p=service&cancer=lung) basing on the Gene Expression Omnibus (GEO) databases GSE29013 and GSE14814, whose information can be retrieved from the internet link https://www.ncbi.nlm.nih.gov/geo/query/acc.cgi?acc=GSE29013 and https://www.ncbi.nlm.nih.gov/geo/query/acc.cgi?acc=GSE14814, respectively.

### Statistical analysis

Statistical significance was assessed using the two tailed Student's T-test or ANOVA with the Bonferroni post-test. Differences were considered statistically significant when P < 0.05. Quantitative data represented the mean ± SEM from at least three independent experiments of biological replicates and were analyzed by using GraphPad Prism software unless otherwise indicated. Figures show representative experiments. Asterisks indicate statistical significance compared to the corresponding control: *, p < 0.05; **, p < 0.01; ***, p < 0.001; and ****, p < 0.0001.

## Results

### ALDH1A1 dependence in erlotinib-resistant lung adenocarcinomas

Erlotinib-resistant lung adenocarcinoma cell lines were generated by chronically exposing cells to escalating erlotinib concentrations of erlotinib as described in the Materials and methods according to our previous reports 20, 21. Ten erlotinib-resistant variants, HCC827-ER1-ER10, were derived from HCC827, an EGFR-activating mutant lung adenocarcinoma cell line. They were >100-fold less sensitive to erlotinib than the parental HCC827 cells (Supplementary Fig. S1A and Table S2). Among them, five resistant cell lines exhibited upregulated ALDH1 activity, compared with the parental HCC827 cells (Supplementary Fig. S1B, C). Pharmacological assay was designed to evaluate the concentration-effect of the ALDH1 inhibitor disulfiram on the inhibition of cell viability (Supplementary Fig. S1D). There was a positive correlation between ALDH1 activity and sensitivity to ALDH1 inhibition in these cells, with HCC827-ER5 being the most sensitive (Supplementary Fig. S1D, E). HCC827-ER5 cells showed substantial upregulation of ALDH1 activity (Supplementary Fig. S1B, C). Reverse transcription-quantitative polymerase chain reaction analysis (RT-qPCR) analysis demonstrated that among ALDH1 subtypes, the 1A1 was significantly upregulated (Supplementary Fig. S1F). The upregulation of ALDH1A1 protein was confirmed by western blot analysis (Supplementary Fig. S1G). Among the erlotinib-resistant variants derived from PC9, another lung adenocarcinoma cell line harboring an EGFR-activating mutation, PC9-ER1 and -ER3 exhibited upregulated ALDH1A1 (Supplementary Fig. S1H-J). PC9-ER3 cells showed greater ALDH1A1 upregulation than PC9-ER1 cells and were more sensitive to ALDH1 inhibition (Supplementary Fig. S1H-K).

**Figure 1:**
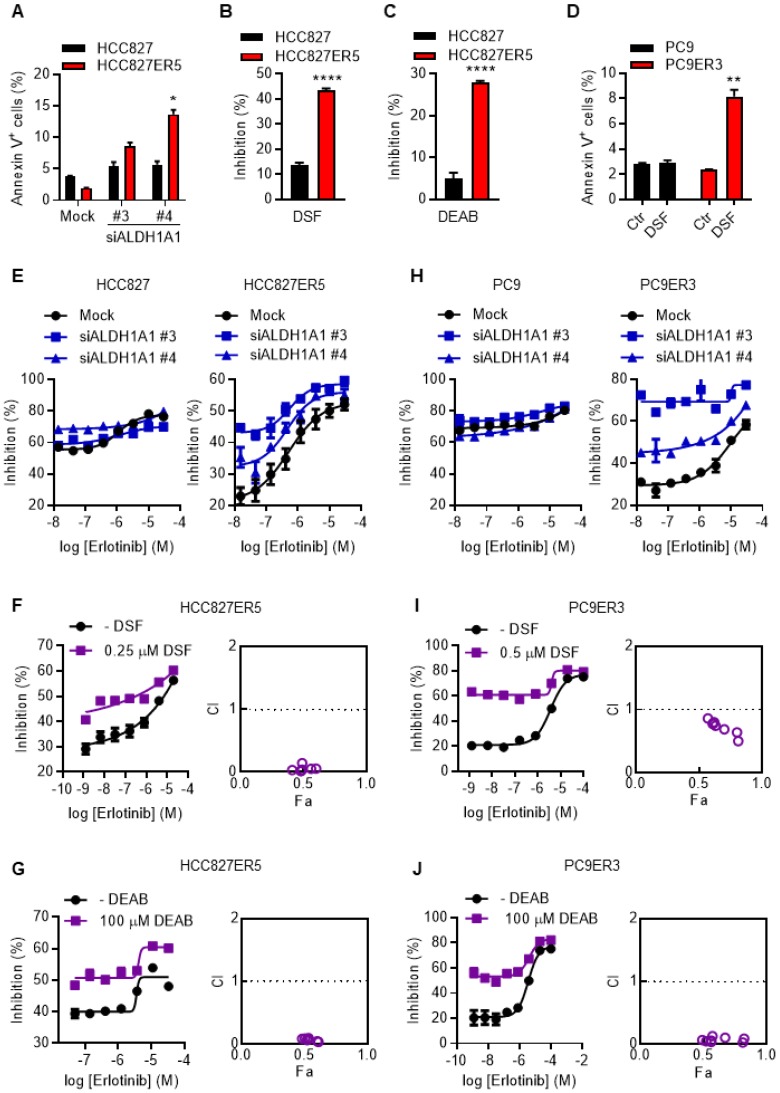
** ALDH1A1 dependence in erlotinib-resistant lung adenocarcinoma cells.** (A) ALDH1^+^ erlotinib-resistant HCC827-ER5 cells were more sensitive to ALDH1A1 knockdown than parental HCC827 cells. The cells were transfected with 30 nM ALDH1A1 siRNA or mock siRNA control for 72 h, stained with PI and FITC-labeled Annexin V, and analyzed by flow cytometry. Annexin V-positive cells indicated the cell undergoing apoptosis. (B-C) HCC827-ER5 cells showed more sensitivity to ALDH1 inhibition analyzed using the CCK8 cell viability assay. The cells were exposed to ALDH1 inhibitors, 1 μM DSF (B) or 100 μM DEAB (C) for 72 h. DSF, disulfiram; DEAB, N,N-diethylaminobenzaldehyde. (D) The ALDH1^+^ erlotinib-resistant PC9-ER3 cells showed more sensitivity to ALDH1 inhibition. The cells were exposed to 1 μM DSF for 24 h. (E and H) Knockdown of ALDH1A1 selectively enhanced the cell viability inhibition effect of erlotinib on HCC827-ER5 (E, right panel) and PC9-ER3 (H, right panel) cells, but not their parental counterparts (E and H, left panels). The cells were exposed to siRNA (30 nM) with erlotinib for 72 h. (F-G) Synergistic effect of ALDH1 inhibition by DSF (F) or DEAB (G) with erlotinib treatment on HCC827-ER5. (I-J) Synergistic effect on PC9-ER3 cells. The cells were co-treated with erlotinib and DSF or DEAB for 72 h. CI value (combination index) was calculated as described in the Materials and methods; CI = 0.85-0.9, slight synergism; CI = 0.7-0.85, moderate synergism; CI = 0.3-0.7, synergism; CI = 0.1-0.3, strong synergism; CI < 0.1, very strong synergism. Data represent the mean ± SEM from at least three independent experiments. In cases where error bars are not apparent, they lie within the space occupied by the symbol.

To examine the dependence of erlotinib-resistant cells on ALDH1A1, apoptosis and viability of the cells were evaluated after genetic or pharmacological suppression of ALDH1A1 by using small interfering RNA (siRNA) or selective inhibitors, respectively. Erlotinib-resistant cells were more sensitive to the suppression of ALDH1A1 compared with their parental counterparts (Fig. 1 A-D). ALDH1A1 knockdown elicited 4-7-fold increased apoptosis in HCC827-ER5 cells versus a ~1.5-fold increase in their parental counterparts (Fig. 1A). Pharmacological blocking of ALDH1 also inhibited HCC827-ER5 cell viability more than that of parental cells (Fig. 1B, C). These selective effects on erlotinib-resistant cells were confirmed in PC9-ER3 cells (Fig. 1D). Moreover, viability assays showed that downregulating or inhibiting ALDH1 re-sensitized the erlotinib-resistant HCC827-ER5 cells (Fig. 1E, right panel; 1F, G) and PC9-ER3 cells (Fig. 1H, right panel; 1I, J) to erlotinib, but did not sensitize the parental cells (left panels in Fig. 1E, 1H). The combination index (CI) was less than 1.0 (right panels in Fig. 1F, G, I, J), demonstrating a synergistic effect of ALDH1 inhibition and erlotinib treatment in erlotinib-resistant cells.

**Figure 2:**
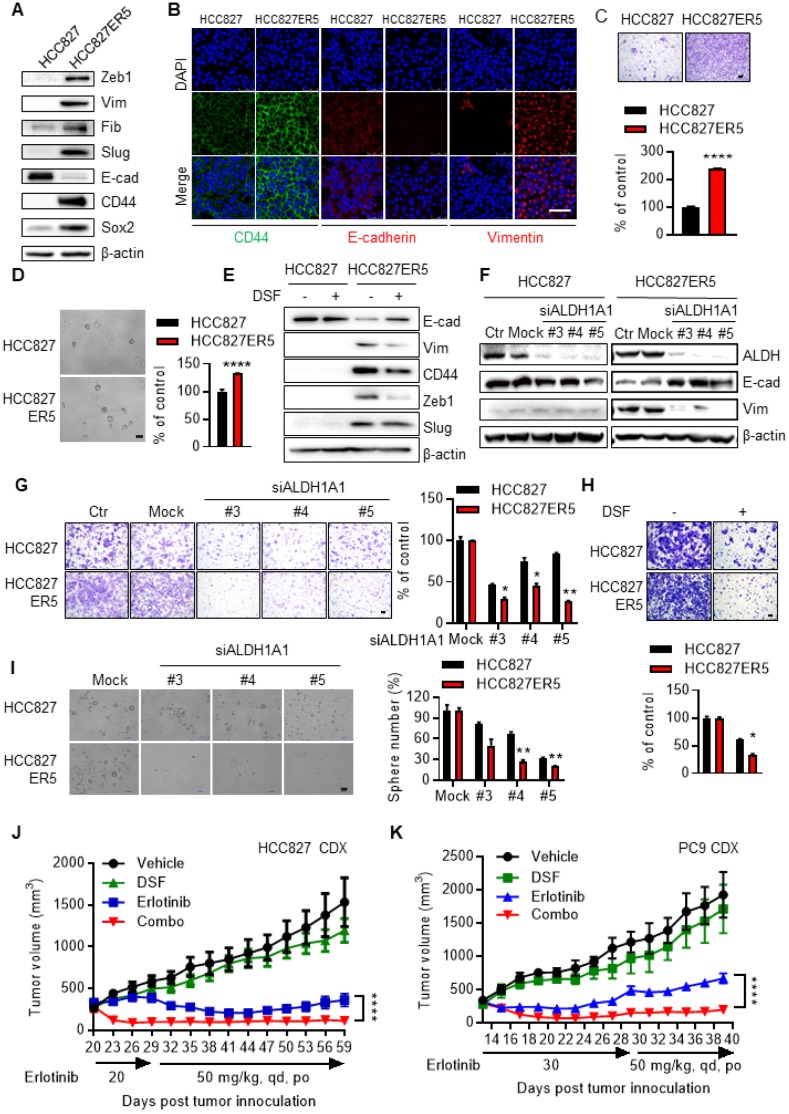
** Elevated EMT/CSC properties of the resistant cells depend on ALDH1A1 and targeting ALDH1 overcomes acquired resistance to erlotinib *in vivo*.** (A) Western blot analysis of mesenchymal markers Zeb1, Vim, Fib, and Slug; epithelial marker E-cad; and cancer stemness markers CD44 and Sox2. Vim, vimentin; Fib, fibronectin; E-cad, E-cadherin. (B) Immunofluorescence staining analysis of CSC/EMT markers. DAPI represents the cell nucleus position. Scale bar: 100 µm. (C) Increased migration ability of erlotinib-resistant cells measured by transwell migration assay. Scale bar: 100 µm. Migration ability of HCC827 cells as control. (D) Increased tumorsphere formation ability of the erlotinib-resistant cells. Scale bar: 100 µm. Tumorsphere formation ability of HCC827 cells as control. (E-F) Pharmacological inhibition (E) or genetic knockdown (F) of ALDH1A1 reversed the CSC/EMT properties of erlotinib-resistant cells assayed by western blot analysis of the markers. The cells were exposed to 1 μM DSF for 48 h or 30 nM siRNA for 72 h. (G-H) Genetic knockdown (G) or pharmacological inhibition (H) of ALDH1A1 reversed the increased migration ability of the resistant cells. After the cells were transfected with 30 nM siRNA or treated with 100 μM DSF for 6 h, the transwell migration assays were performed in fresh media without the siRNA or inhibitor. Mock (G) or DSF free (H) data of each corresponding cell line as control. (I) Elevated sphere formation of erlotinib-resistant cells was more sensitive to the knockdown of ALDH1A1. The cells were transfected with 30 nM siRNA for 72 h, and then the sphere formation was performed in the medium without the siRNA. (J and K) Inhibition of ALDH1 overcomes the acquired resistance to erlotinib in HCC827 and PC9 cell-derived xenograft tumors. For the drug treatment group, xenograft harboring mice were treated with the indicated dosage of erlotinib, DSF (60 mg/kg, qd, po), or their combination. The growth of tumors was monitored every 2 d. Tumor (2 per mouse) volume and mouse body weight are presented as mean ± SEM from five mice per group. Details for the xenograft assay are described in Materials and methods.

The ALDH-upregulated erlotinib-resistant cells acquired CSC-like/EMT properties. HCC827-ER5 cells showed a spindle-like shape, including the loss of apical-basal polarity and cell-cell contacts, in contrast to their parental counterparts which exhibited an epithelial morphology (Supplementary Fig. S2A). Moreover, the HCC827-ER5 cells showed upregulated expression of the CSC markers CD44 and Sox2 (Fig. 2A, B) and mesenchymal markers vimentin, Zeb1, fibronectin, and slug (Fig. 2A, B), and decreased expression of the epithelial marker E-cadherin (Fig. 2A, B). These cells also demonstrated an increased ability of migration (Fig. 2C), an EMT hallmark, and of tumorsphere formation, a hallmark of CSCs *ex vivo* (Fig. 2D). Pharmacological inhibition and RNA interference of ALDH1 reversed the elevated CSC/EMT properties in the erlotinib-resistant cells, as shown by the selective regulation of epithelial and mesenchymal markers (Fig. 2E, F), migration (Fig. 2G, H), and tumorsphere formation (Fig. 2I). The increased EMT/CSC properties of ALDH-upregulated, erlotinib-resistant cells were further confirmed in PC9-ER3 cells (Supplementary Fig. S2B-D). ALDH1A1 suppression also reversed the elevated CSC/EMT properties in these cells (Supplementary Fig. S2E, F). The erlotinib-resistant HCC827-ER5 and PC9-ER3 cells demonstrated increased levels of phosphorylated pEGFR and pAKT (Supplementary Fig. S2G). Erlotinib, though resisted by these cells, efficiently blocked EGFR singling in these cells, demonstrating substantially decreased levels of pAKT, pERK, and pEGFR (Supplementary Fig. S2G). ALDH1A1 suppression, though significantly inhibited erlotinib-resistant cells (Fig. 1A-D; Supplementary Fig. S1D, K), did not by itself block EGFR signaling in these cells (Supplementary Fig. S2G). These data imply that ALDH1A1 suppression inhibits erlotinib-resistant cells in a mechanism other than EGFR signaling.

These data indicate that the erlotinib-resistant cancer cells depended on and were addicted to ALDH1A1 upregulation, and that they were sensitive to ALDH1-targeting treatments. Moreover, suppression of ALDH1A1 reversed CSC/EMT and overcame erlotinib resistance in these cells.

The observation that targeting ALDH1 overcomes erlotinib resistance was recapitulated in erlotinib-induced acquired resistance models *in vivo*. HCC827 (Fig. 2J)- and PC9 (Fig. 2K)-cell derived xenograft (CDX) tumors initially responded to erlotinib, showing decreased tumor volumes after drug treatment. The effect of erlotinib on tumor growth inhibition maximized at day 41-44 and day 21-23 in HCC827- and PC9-CDX models, respectively (Fig. 2J, K). However, continuous erlotinib administration resulted in the gradual acquisition of drug resistance in the tumors, showing a relapse of the tumor burden (Fig. 2J, K). While the tumors gradually acquired resistance to single-agent erlotinib, they showed much greater sensitivity to combined treatment with erlotinib and the ALDH1 inhibitor disulfiram in both HCC827- and PC9-CDX mouse models (Fig. 2J, K), even demonstrating a complete recession of the tumors (Fig. 2J, K and Supplementary Fig. S3A, B). The hematoxylin and eosin (H&E) staining analysis showed DSF and the combination treatment induced tumor necrosis (Supplementary Fig. S3C). Western blot analysis of the xenograft tumors demonstrated that the effect of ALDH1 inhibition correlated with the expression levels of ALDH1A1 (Supplementary Fig. S3D). Moreover, inhibition of ALDH1 tended to block tumor metastasis to the brain (Supplementary Fig. S3E) There was no notable toxicity measured on the basis of body weight change when disulfiram was combined to overcome the acquired resistance to erlotinib (Supplementary Fig. S3F).

Drug-resistant cells, owing to their more quiescent CSC/EMT phenotype, characterized by prominent metastasis, the major contributor to cancer-related death, rather than by local tumor formation *in vivo* 24-26. We then further examined if ALDH1 inhibition overcame the erlotinib resistance-induced metastasis and the subsequent early death *in vivo*. The mice transplanted with the erlotinib-resistant cells, compared to those with the parental cells, died much earlier (HCC827ER5 vs HCC827 and PC9ER3 vs PC9, vehicle control group, in Supplementary Fig. S3G-J). Pathological analysis demonstrated these mice bore substantial disseminated tumor foci in lungs and livers (Supplementary Fig. S4A-F). ALDH1 inhibition by DSF markedly prolonged mouse survival (Supplementary Fig. S3G-J) and suppressed tumor dissemination both in HCC827-ER5 and PC9-ER3 models (Supplementary Fig. S4A-F), even without combination with erlotinib, indicating its prominent effect on erlotinib-resistant tumors. These results were very consistent with our *in vitro* observations that the enhanced EMT/CSC properties and migration ability of the erlotinib-resistant cells were substantially abrogated by ALDH1 suppression (Fig. 2E, G, H, I; Supplementary Fig. S2E, F).

### ALDH1A1 induces resistance to erlotinib in lung adenocarcinomas

**Figure 3:**
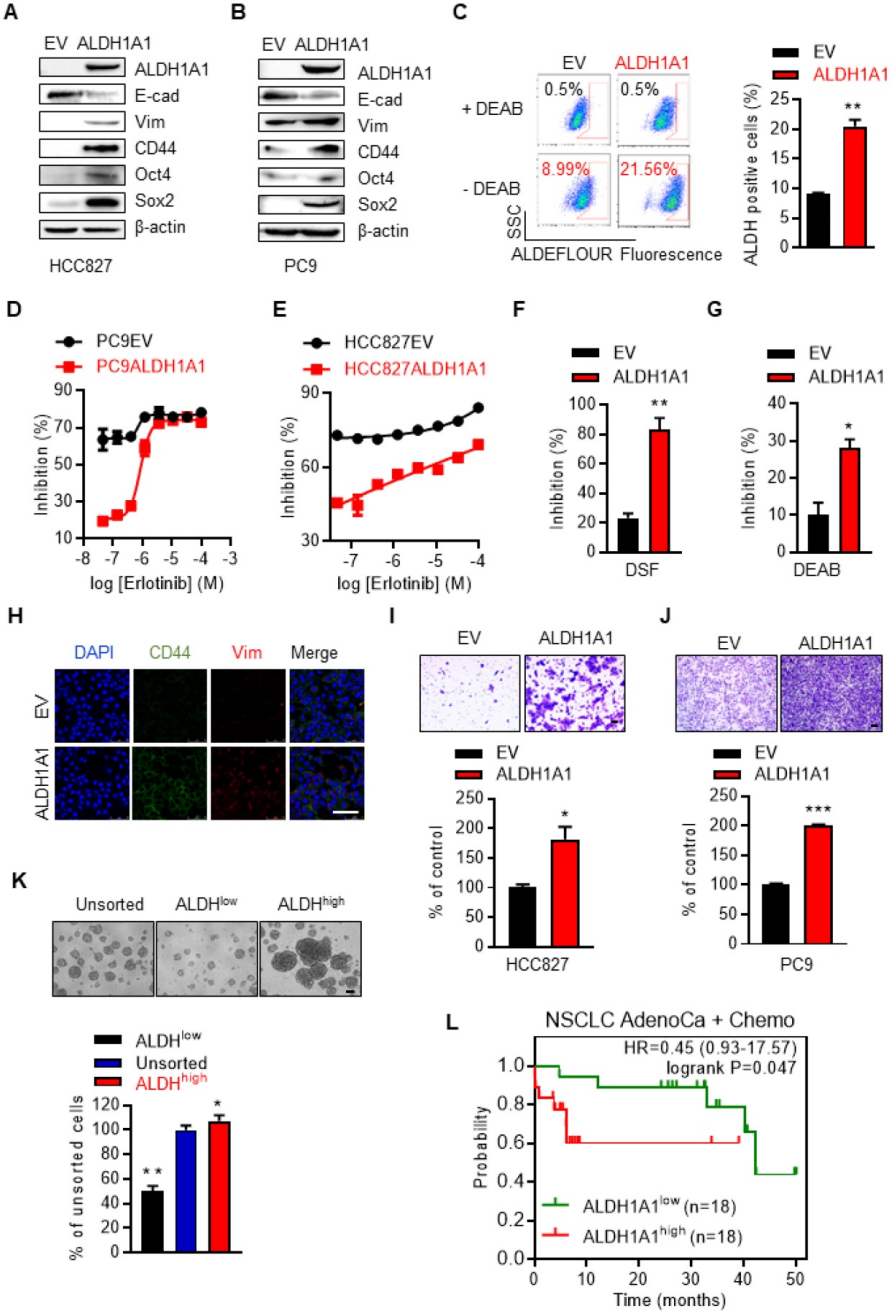
** ALDH1A1 induces resistance to erlotinib in lung adenocarcinomas.** (A-B) Western blot analysis of ecto-expression of ALDH1A1 in HCC827 and PC9 cells. The cells were infected with retroviruses expressing ALDH1A1 or empty vectors (EV) as control. (C) Analysis of ALDH1^+^ cells in HCC827-EV and HCC827-ALDH1A1 cells measured by Aldefluor assay. (D-E) Ecto-expression of ALDH1A1 decreased the inhibitory effect of erlotinib on PC9 (D) and HCC827 (E) cells analyzed by CCK8 cell viability assay. The cells were exposed to erlotinib for 72 h. (F-G) HCC827-ALDH1A1 cells were more sensitive to ALDH1 inhibition by DSF (F) and DEAB (G) compared with the control cells analyzed by CCK8 cell viability assay. The cells were exposed to 10 μM DSF or 100 μM DEAB for 72 h. (H) ALDH1A1 ecto-expression induced CSC/EMT properties assayed by immunofluorescence analysis. Scale bar: 100 µm. (I-J) ALDH1A1 ecto-expressed HCC827 (I) and PC9 (J) cells acquired increased migration ability analyzed by transwell migration assay. Scale bar: 100 µm. (K) Sphere formation assay of FACS-sorted ALDH^+^ and ALDH^-^ cells. Scale bar: 100 µm. (L) Kaplan-Meier analysis of the association between the probability of overall survival (OS) of lung adenocarcinoma patients who were received chemotherapy (n=36) and their ALDH1A1 gene expression profiles. The analysis was performed by using the online KM-plotter tool (http://kmplot.com/analysis/index.php?p=service&cancer=lung) basing on the Gene Expression Omnibus (GEO) databases GSE29013 and GSE14814. Low or high levels of ALDH1A1 was defined as higher or lower than the median value of 36 patients.

We then examined whether ALDH1A1 upregulation was sufficient to induce erlotinib resistance. Ectopic expression of ALDH1A1 (Fig. 3A, B) increased the percentage of cells with high ALDH1 activity (Fig. 3C). This allowed the otherwise sensitive cells to withstand exposure to erlotinib, showing a right shift of erlotinib concentration-cell viability inhibition effect curves in PC9 and HCC827 cells (Fig. 3D, E). These cells, as gradually addicted to ALDH1A1, were sensitive to ALDH1 suppression (Fig. 3F, G) as expected. The CSC markers CD44, Oct4, and Sox2, and the mesenchymal markers vimentin and Zeb1 were upregulated, while the epithelial marker E-cadherin was downregulated following ALDH1A1 overexpression (Fig. 3A, B, H). ALDH1A1 overexpression also facilitated cell migration (Fig. 3I, J). Moreover, ALDH^+^ cells isolated by florescence-activated cell sorting (FACS) showed increased tumorsphere-formation ability, compared with ALDH^-^ cells (Fig. 3K). These data indicate that ALDH1A1 upregulation led to CSC/EMT properties and erlotinib resistance in lung adenocarcinoma cells. In parallel, in patients undergoing chemotherapy, those with ALDH1A1 upregulation showed lower overall survival rates (Fig. 3L).

### ALDH1A1-addicted erlotinib-resistant cells evolve an enhanced anti-ROS-RCS system

**Figure 4:**
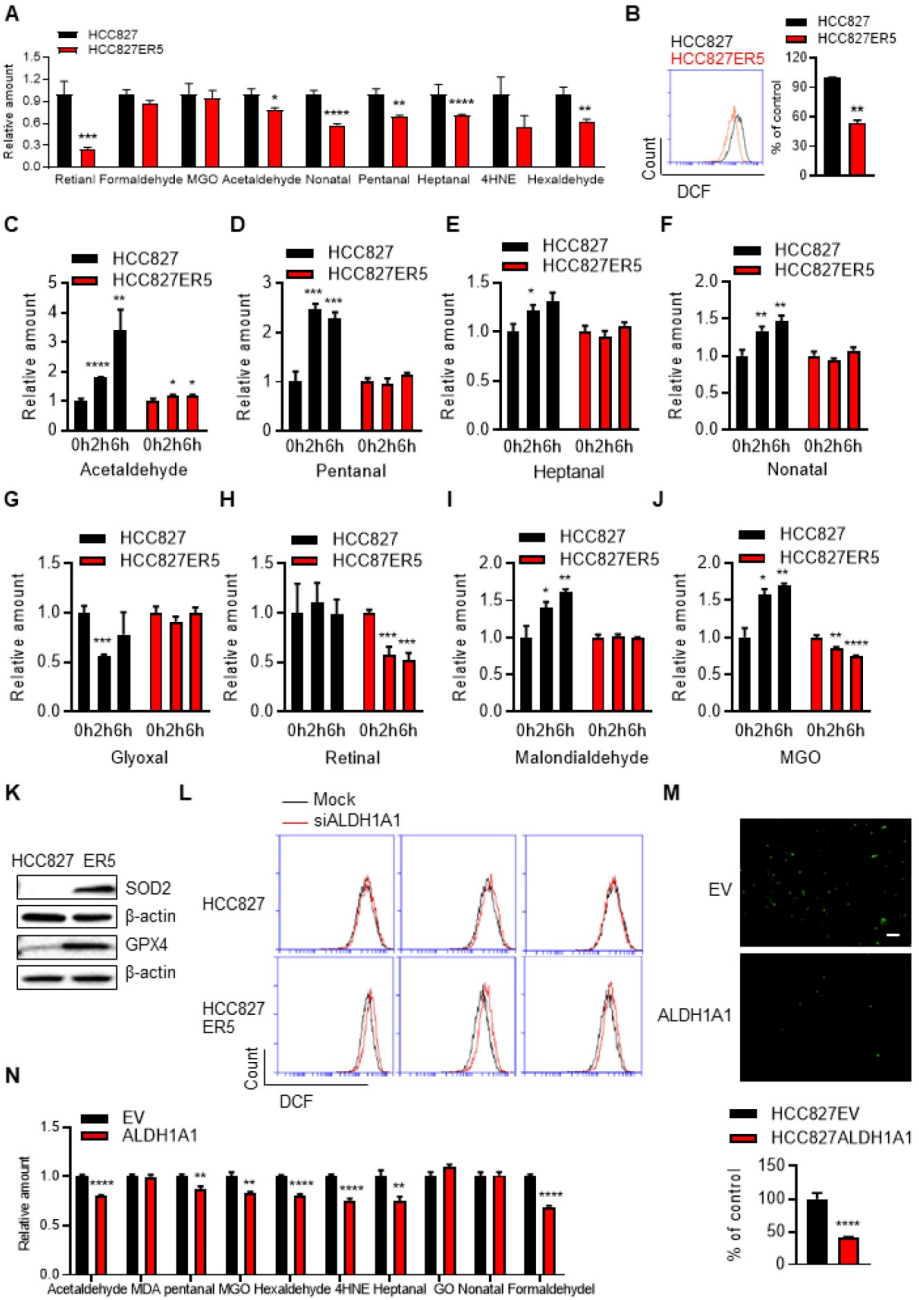
** ALDH1A1-addicted erlotinib-resistant cells evolve an elevated anti-ROS/RCS system.** (A-B) Lower levels of intracellular RCS (A) and ROS (B), analyzed by LC-QqQ-MS/MS and flow cytometry, respectively, in HCC827-ER5 cells compared to their parental counterparts. MGO, methylglyoxal; 4HNE, 4-hydroxy-2-nonenal. (C-J) HCC827-ER5 cells were resistant to erlotinib-induced intracellular RCS accumulation compared to the parental cells. The RCS were detected by LC-QqQ-MS/MS. The cells were treated with 1 μM erlotinib. (K) Upregulation of RCS and ROS mitigating enzymes, GPX4 and SOD2, respectively, in HCC827-ER5 cells assayed by western blot analysis. (L) Knockdown of ALDH1A1 induced ROS accumulation, more obviously in HCC827-ER5 cells than in their parental cells. The mock effect was shown as the curve of the black solid line compared with the treatment effect shown as the red. The cells were transfected with ALDH1A1 or control siRNA for 48 h and stained with DCFH-DA for flow cytometry analysis. (M-N) Overexpression of ALDH1A1 decreased the intracellular levels of ROS (M) and RCS (N). The cells were stained with DCFH-DA and imaged and analyzed *in situ* using an IncuCyte living cell cytometer.

Untargeted transcriptomic and metabolomic analysis of ALDH1A1-addicted, erlotinib-resistant cells demonstrated a robust change in the glutathione (GSH) and nicotinate & nicotinamide metabolism pathways (Supplementary Fig. S5A), which are closely linked to ROS-RCS metabolic pathways. Then, more precise targeted analyses were performed. RCS were detected by selective-reaction monitoring (SRM) of liquid chromatography coupled to triple-quadrupole tandem mass spectrometry (LC-QqQ-MS/MS) and ROS were determined by flow cytometric and *in situ* cell imaging analyses based on detecting fluorescent ROS probes. Compared with their erlotinib-sensitive parental counterparts, the resistant cells generally bore lower basal RCS levels (Fig. 4A). The total intracellular ROS (Fig. 4B) and mitochondrially generated ROS levels were also lower in these resistant cells (Supplementary Fig. S5B). Under acute erlotinib challenge, RCS were induced and accumulated in erlotinib-sensitive cells, but not in resistant cells (Fig. 4C-J). In agreement, key enzymes involved in metabolizing RCS and ROS (GPX4 and SOD2, respectively), as well as ALDH1A1, were upregulated in the resistant cells (Fig. 4K and Supplementary Fig. S5C). ALDH1A1 knockdown induced ROS accumulation and showed more obviously in resistant cells (Fig. 4L and Supplementary Fig. S5D). Moreover, DOX-induced ALDH1A1 expression decreased the levels of ROS (Fig. 4M and Supplementary Fig. S5E-H) and RCS (Fig. 4N) in sensitive parental cells. We then investigated the effect of ROS-RCS metabolic system on erlotinib resistance.

### ALDH1A1-addicted erlotinib-resistant cells depend on the ROS-RCS metabolic pathway

**Figure 5:**
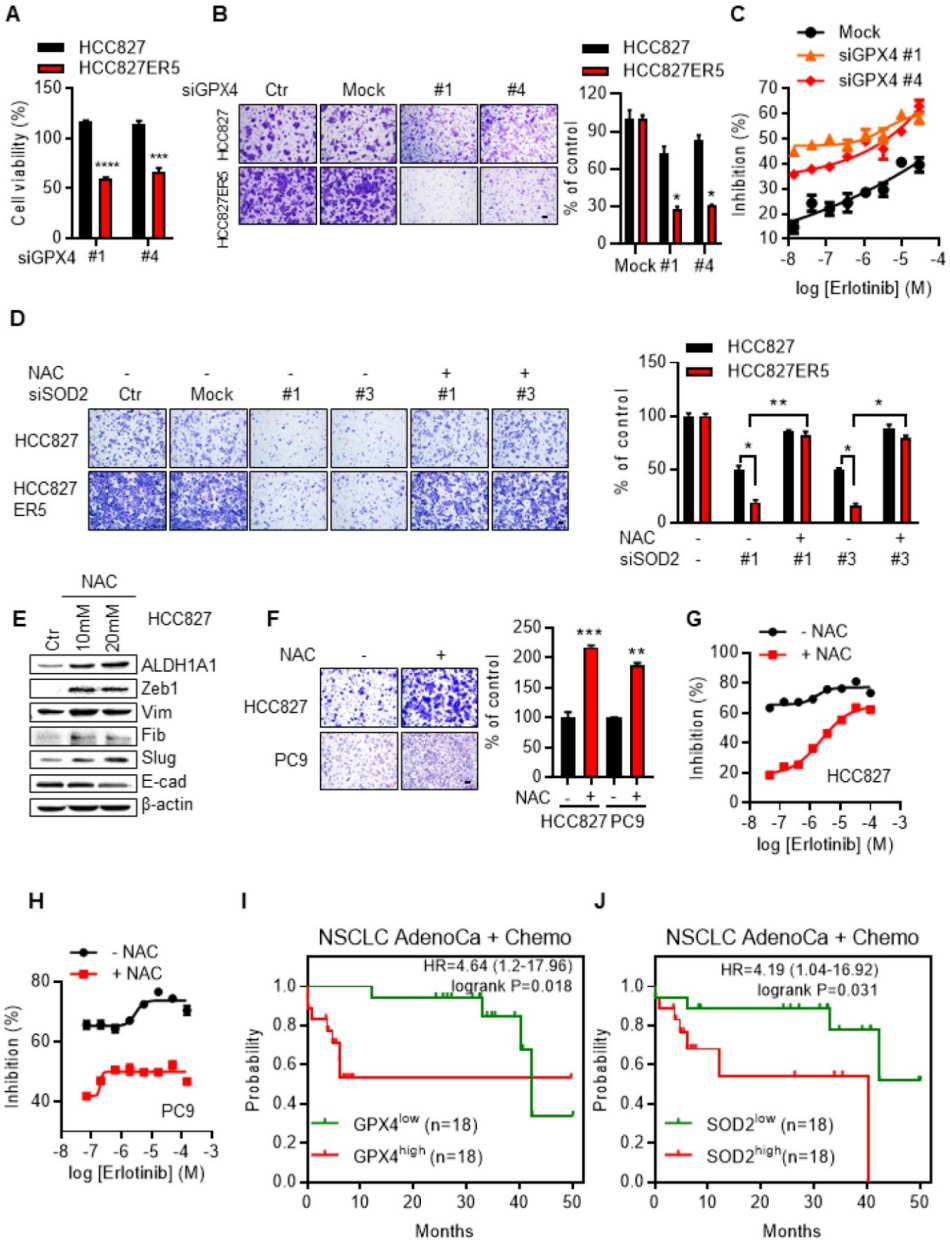
** ALDH1A1-addicted erlotinib-resistant cells depend on ROS-RCS metabolic pathway.** (A-B) Knockdown of RCS mitigating enzyme GPX4 selectively inhibited HCC827-ER5 cell viability (A) and migration (B). The cells were transfected with siRNA for 72 h. (C) GPX4 knockdown re-sensitized HCC827-ER5 cells to erlotinib-induced inhibition of cell viability. The cells were transfected with siRNA for 72 h. (D) Knockdown of SOD2 selectively abrogated the elevated migration in HCC827-ER5 cells, and the effect was reversed by ROS and RCS scavenger N-acetyl-L-cysteine (NAC). The cells were transfected with SOD2 or mock control siRNA for 72 h. NAC (10 mM) was added 6 h before the point of the migration measurement. (E-F) Scavenging of ROS-RCS induced EMT properties assayed by mesenchymal/epithelial marker analysis (E) and migration ability analysis (F). The cells were exposed to 10 or 20 mM (E) or 10 mM (F) NAC for 6 h. (G-H) Scavenging ROS-RCS rendered HCC827 and PC9 parental cells less sensitive to erlotinib. The cells were exposed to 10 mM NAC for 6 h. (I-J) Kaplan-Meier analysis of the association between the probability of overall survival (OS) of lung adenocarcinoma patients who were received chemotherapy (n=36) and their GPX4 (I) and SOD2 (J) gene expression profiles. The analysis was performed by using the online KM-plotter tool (http://kmplot.com/analysis/index.php?p=service&cancer=lung) basing on the Gene Expression Omnibus (GEO) databases GSE29013 and GSE14814. Low or high levels of gene were defined as higher or lower than the median value of 36 patients. Ctr, solvent control; NSCLC, non-small-cell lung carcinoma; AdenoCa, adenocarcinoma; Chemo, with chemotherapy.

Knockdown of the RCS-mitigating enzyme GPX4 selectively inhibited both the viability (Fig. 5A) and the upregulated migration ability (Fig. 5B and Supplementary Fig. S6A) of ALDH1A1-addicted resistant cells. The selective inhibition effect was abrogated by the GSH precursor, ROS-RCS scavenger N-acetyl-L-cysteine (NAC), confirming that the effect depended on the ROS-RCS metabolic pathway (Supplementary Fig. S6A). Moreover, GPX4 knockdown re-sensitized erlotinib-resistant cells to erlotinib-induced inhibition of cell viability (Fig. 5C and Supplementary Fig. S6B). SOD2 Knockdown selectively re-sensitized the erlotinib-resistant cells to erlotinib (Supplementary Fig. S6C) and abrogated the elevated migration of the resistant cells (Fig. 5D and Supplementary Fig. S6D). Although SOD2 knockdown also inhibited the parental cell migration (~50%), the inhibition effect was more obvious in resistant cell (~83%) as shown in Fig. 5D. The effect of abrogation of the elevated migration in resistant cells was reversed by NAC (Fig. 5D and Supplementary Fig. S6D), confirming its dependence on the ROS-RCS metabolic pathway.

In contrast to the above demonstration that activating ROS-RCS system by knockdown of SOD2 or GPX4 overcame erlotinib resistance, ROS-RCS scavenging induced the parental cells that were otherwise sensitive to erlotinib to acquire EMT and the ability to resist erlotinib. Under this condition, the mesenchymal markers were upregulated, the epithelial markers were downregulated (Fig. 5E), the migration ability increased (Fig. 5F), and the cells became less sensitive to erlotinib (Fig. 5G, H). In parallel with these findings, in patients undergoing chemotherapy, those with GPX4 or SOD2 upregulation had a lower probability of survival (Fig. 5I, J).

### ALDH1A1-conferred erlotinib resistance depends on the ROS-RCS metabolic pathway

**Figure 6:**
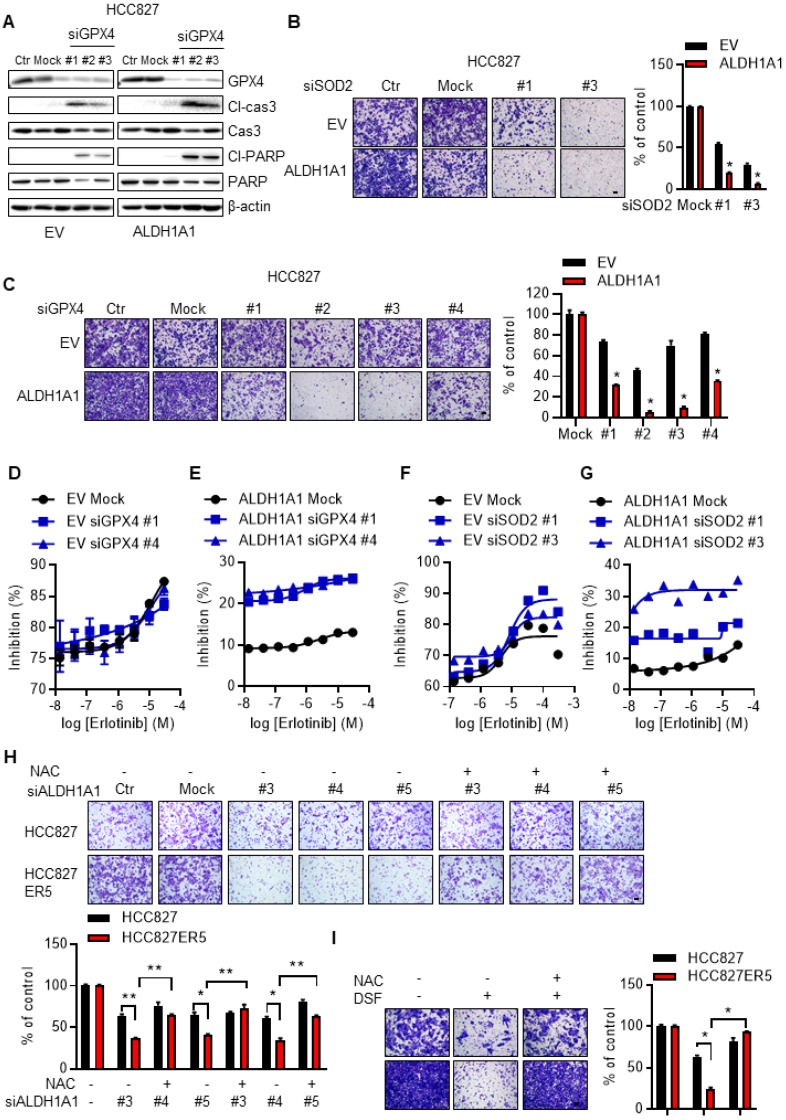
** ALDH1A1-conferred resistance to erlotinib depends on the ROS/RCS metabolic pathway.** (A) Knockdown of GPX4 induced apoptosis more in HCC827-ALDH1A1 than in the control cells, assayed by western blot analysis of the apoptotic markers, cleaved caspase 3 (Cl-cas3) and cleaved PARP (Cl-PARP). The cells were transfected with 20 nM GPX4 siRNA for 72 h. (B-C) Knockdown of SOD2 (B) or GPX4 (C) abrogated the ALDH1A1-induced effect on the enhancement of cell migration ability. The cells were transfected with 20 nM GPX4 or SOD2 siRNA for 72 h. Mock data of each corresponding cell line as control. (D-G) Knockdown of GPX4 (D and E) or SOD2 (F and G) sensitized the effect of erlotinib-induced cell viability inhibition more in HCC827-ALDH1A1 (E and G) than in the control (D and F) cells. The cells were transfected with 20 nM GPX4 or SOD2 siRNA for 72 h. (H-I) Suppression of ALDH1A1 by siRNA (H) or inhibitor (I) abrogated the enhanced migration ability of HCC827-ER5 cells, and the ALDH1A1-mediated effect was reversed by NAC. The cells were transfected with ALDH1A1 or mock control siRNA for 72 h, or exposed to 100 μM DSF for 6 h with or without 10 mM NAC for 6 h, and the migration ability was measured in fresh media without the above-mentioned reagents. Mock (H) or DSF/NAC free (I) data of each corresponding cell line as control.

We then examined the effect of the ROS-RCS metabolic system on ALDH1A1-induced phenotypic changes. The ALDH1A1 ecto-expressed cells that acquired CSC/EMT properties and resisted erlotinib (Fig. 3A-J), were more sensitive to knockdown of the RCS-mitigating enzyme GPX4, showing higher levels of the apoptotic markers cleaved caspase 3 and cleaved PARP (Fig. 6A). In addition, knockdown of SOD2 or GPX4 abrogated the ALDH1A1-induced enhancement of cell migration ability (Fig. 6B, C).

Following GPX4 or SOD2 knockdown, ectopic ALDH1A1-expressing cells, which demonstrated erlotinib resistance, were selectively re-sensitized to erlotinib (Fig. 6D-G). Consistent with these data, activating the ROS-RCS system by suppressing GSH biosynthesis with buthionine sulfoximine (BSO), a selective inhibitor of γ-glutamylcysteine synthetase 27, 28, re-sensitized the erlotinib-resistant cells to erlotinib (Supplementary Fig. S6E). BSO treatment also abrogated ALDH1A1-induced enhancement of the cell migration ability (Supplementary Fig. S6F). In contrast, the effect of suppressing ALDH1A1, i.e., abrogating the enhanced migration ability of erlotinib-resistant cells, was reversed by the ROS-RCS scavenger NAC (Fig. 6H, I).

### RCS-ROS metabolic enzymes are activated by ALDH1A1 and ALDH1A1 confers resistance to erlotinib via the ROS-RCS metabolic pathway *in vivo*

**Figure 7:**
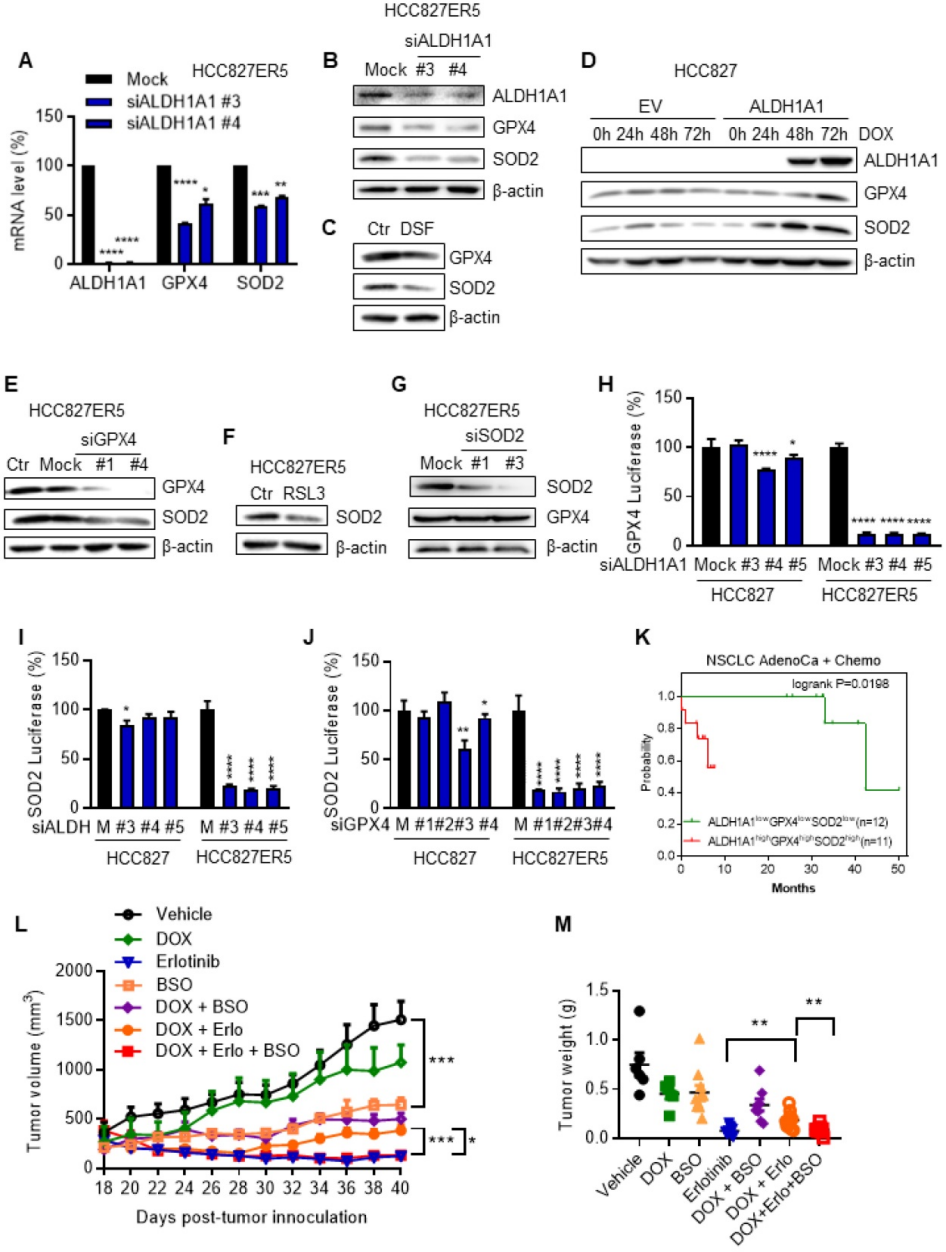
** The RCS**-**ROS metabolic enzymes are activated by ALDH1A1 and ALDH1A1 confers resistance to erlotinib via the ROS**-**RCS metabolic pathway *in vivo*.** (A-C) ALDH1A1 suppression downregulated GPX4 and SOD2 mRNA (A) and protein (B and C) expression levels by treatment with the siRNA (A and B) or the inhibitor (C). The cells were transfected with ALDH1A1 or control mock siRNA for 72 h or treated with 100 μM DSF for 6 h. (D) Conditional induction of ALDH1A1 ecto-expression in HCC827 cells upregulated GPX4 and SOD2 assayed by western blot analysis. DOX, doxycycline. (E-F) SOD2 was downregulated by knockdown or inhibition of GPX4 via siRNA (E) or inhibitor RSL3 (F), respectively, in HCC827-ER5 cells. The cells were transfected with 20 nM GPX4 or mock control siRNA for 72 h, or 100 nM RSL3 for 6 h. (G) Analysis of GPX4 protein levels after SOD2 knockdown in HCC827-ER5 cells. The cells were transfected with 20 nM SOD2 or mock control siRNA for 72 h. (H-I) Knockdown of ALDH1A1 suppressed the promoter activity of GPX4 (H) and SOD2 (I), and knockdown of GPX4 suppressed the promoter activity of SOD2 (J) as analyzed by the dual-luciferase assay for target gene promoter activity. The cells were transfected with 20 nM siRNA for 48 h. M, mock. (K) Kaplan-Meier analysis of the association between the probability of overall survival (OS) of lung adenocarcinoma patients who were received chemotherapy and their ALDH1A1, GPX4, and SOD2 gene co-expression profiles. The analysis was performed by using the online KM-plotter tool (http://kmplot.com/analysis/index.php?p=service&cancer=lung). Low or high levels of genes in tumor samples were defined as higher or lower than the median value of 36 patients. In KM-plotter tool database, the number of lung adenocarcinoma patients who were received chemotherapy is 36. The number of patients whose tumors displayed high expression of all ALDH1A1, GPX4, and SOD2 is 12 and the number of patients with low expression of all three genes is 11. (L-M) DOX-induced ALDH1A1 conferred HCC827 cell-derived xenograft tumor resistance to erlotinib, and this effect was abrogated by GSH synthesis inhibitor BSO. ALDH1A1 in the cells was induced by the Tet-On system for doxycycline-inducible gene expression as described in the Materials and Methods. The mice with subcutaneously implanted tumors were treated with erlotinib (30 mg/kg, qd, po), doxycycline (50 mg/kg, qd, po), BSO (450 mg/kg, qod, ip), or their combinations as indicated. The weight of resected tumors was measured at the end of the experiments. Tumor volumes (2 per mouse) are presented as the mean ± SEM from five mice per group. CTR, vehicle control; Erlo, erlotinib; DOX, doxycycline.

Since the ALDH1A1-addicted, erlotinib-resistant cells were endowed with an upregulated RCS-ROS metabolic ability to deal with drug induced stresses, we further examined the mechanism underlying this upregulation. ALDH1A1 suppression downregulated GPX4 and SOD2 mRNA and protein levels (Fig. 7A-C) in ALDH1A1-addicted, erlotinib-resistant cells where GPX4 and SOD2 were otherwise upregulated (Fig. 4K and Supplementary Fig. S5C). Consistent with these findings, conditional ectopic induction of ALDH1A1 expression in parental HCC827 cells upregulated GPX4 and SOD2 (Fig. 7D). In addition, GPX4 knockdown or inhibition downregulated SOD2 in HCC827-ER5 cells (Fig. 7E, F), while SOD2 knockdown did not change GPX4 protein expression levels in these cells (Fig. 7G). These results suggest that SOD2 was regulated by GPX4, which was controlled by ALDH1A1. This hypothesis was confirmed by performing dual-luciferase assays to test target-gene promoter activities. ALDH1A1 knockdown suppressed the promoter activities of GPX4 and SOD2 (Fig. 7H, I) and GPX4 knockdown suppressed the promoter activity of SOD2 (Fig. 7J). The effects of the promoter activity inhibition by the knockdown of ALDH1A1 or GPX4 were more substantial in ALDH1A1-addicted, erlotinib-resistant cells (Fig. 7H-J). These data indicate that the RCS-ROS-metabolic pathway was controlled by the ALDH1A1-GPX4-SOD2 axis in ALDH1A1-addicted, erlotinib-resistant cells. In parallel with these findings, in patients undergoing chemotherapy, those with high co-expression of ALDH1A1, GPX4, and SOD2 had a lower probability of survival than the patients with low expression of these three molecules (Fig. 7K).

To verify the ROS-RCS metabolic-dependence mechanism in ALDH1A1-induced resistance *in vivo*, we examined whether activating the ROS-RCS system, by suppressing GSH biosynthesis with BSO 27, 28, would abrogate ALDH1A1-induced erlotinib resistance in a human CDX mouse model. Conditional induction of ALDH1A1 gradually conferred resistance to erlotinib, showing a lower response of the doxycycline (DOX)-induced ALDH1A1 group to erlotinib compared to the group without ALDH1A1 induction under erlotinib treatment (Fig. 7L, M); this effect was abrogated by BSO treatment (Fig. 7L, M).

## Discussion

Rewired metabolism has recently been regarded as a core hallmark of cancer. However, whether or how cancer cells further reprogram the metabolism to acquire resistance to molecular targeted drugs is poorly understood. Here we find a previously unidentified mechanism by which ALDH1A1 confers erlotinib resistance by facilitating the ROS-RCS metabolic pathway in lung adenocarcinomas.

ALDH1A1 is a key CSC marker and EMT inducer 3, 29, 30. Its expression levels inversely correlate with the prognosis of various tumors in retrospective clinical analyses, whereas in prospective studies, upregulated ALDH1A1 levels predicted a poor prognosis 7, 31-33. Drug-resistant cells often bear activated and upregulated ALDH1A1, representing an important mechanism whereby tumors can resist drug treatment 3, 9, 34, 35.

We found that ALDH1A1 was upregulated in a subset of erlotinib-resistant cells; this upregulation led to a stem cell-like properties and EMT, endowing cell with the ability to withstand erlotinib drug stress, while making these cells dependent on and addicted to ALDH1A1. Targeting ALDH1A1 reversed the acquired CSC/EMT properties and overcame erlotinib resistance; moreover, pharmacological inhibition of ALDH1 re-sensitized the tumors to erlotinib and abrogated erlotinib resistance *in vitro* and *in vivo*.

These results indicate that targeting ALDH1 may be a promising clinical strategy for overcoming TKI resistance. ALDH1 is upregulated in circulating tumor cells and acts as a biomarker for detecting and isolating these cells in patients with cancer recurrence, and its levels are predictive of responses of drug therapy 36, making it possible to target ALDH1-dependent cancers more precisely.

Mechanistically, we found that ALDH1A1 exerted its effects by facilitating the RCS-ROS metabolic system. Cancer cells bear higher ROS and RCS tension, which renders them closer to the death threshold of oxidative/carbonyl stress, underpinning important mechanisms of the anticancer effects of conventional cytotoxic drugs and new molecularly targeted agents 14, 37, 38. When challenged by anticancer treatment, intracellular ROS levels are induced and accumulate. The increased levels of ROS are sustained, until the early stage of an adaptive tolerant response 39. Compared with the reversibly adaptive tolerant stage 21, 40, 41 where the antioxidant system has not yet sufficiently evolved to counteract elevated ROS levels 39, at the acquired resistance stage 21, 40, 41, ALDH1A1-addicted cells maintain lower levels of RCS/ROS owing to their ability to upregulate the anti-RCS/ROS system. In agreement, isolated and enriched ALDH^+^ breast and ovarian cancer cells have much lower levels of ROS 13, 42.

Cancer cells depend on an antioxidant system that defends against oxidative and carbonyl stress to survive. It is known that CSC/EMT and therapy-resistant cells can acquire tolerance to drug stress by upregulation of that protective system 12, 14, 15. We found that the inductive effects of ALDH1A1 on CSC/EMT and erlotinib resistance depended on modulation of the RCS-ROS metabolic pathway. The ALDH1A1-addicted, erlotinib-resistant cells bore upregulated levels of GPX4 and SOD2, as well as ALDH1A1, and maintained lower basal levels of RCS and ROS. By analogy, some cancer cells can acquire drug resistance via CD44- or NRF2-mediated GSH upregulation and ROS scavenging, or by upregulating RCS-metabolizing enzymes 43, 44.

SOD2 is a pivotal antioxidant enzyme that dismutates superoxide radicals to oxygen and hydrogen peroxide, which can be degraded metabolically. SOD2 upregulation promoted cancer cell migration, invasion, and stemness, and conferred drug resistance, whereas SOD2 inhibition increased ROS levels and substantially impeded cancer progression *in vitro* and *in vivo* 45. Drug-induced ROS can initiate RCS accumulation, mainly through the oxidative degradation of lipids. Behaving as a biomarker of oxidative damage, elevated RCS levels can injure cells and further exacerbate ROS accumulation, forming a mutually amplifying cycle 16, 46. Indeed, cancer cells are inhibited or killed not only by ROS, but also by RCS 18, 47, 48. GPX4 (phospholipid hydroperoxidase), which differs from the other glutathione peroxidase family members that mainly reduce free hydrogen peroxide, is the only peroxidase capable of selectively reducing lipid hydroperoxides and sufficiently decreasing subsequent RCS accumulation as ALDH 49. This unique characteristic enables GPX4 pivotal in resisting lipid peroxidation-dependent cell death 49, 50. Moreover, cancer cells with mesenchymal chemotherapy-resistant properties depend on GPX4 for survival, and they are more sensitive to GPX4 suppression 51, 52. Targeting GPX4 represents a potential strategy for overcoming drug resistance 51, 52.

We found that ALDH1A1 regulated the SOD2 and GPX4 gene promoters. In parallel, GPX4 expression positively correlated with elevated ALDH1A1 levels in colon CSCs 53, and fluorescence-activated cell sorting (FACS) analysis revealed that SOD2 was upregulated in ALDH^+^ ovarian cancer cells 42.

In summary, we found that erlotinib-resistant lung adenocarcinoma cells depended on ALDH1A1 and that ALDH1A1 conferred EMT and drug resistance by facilitating the ROS-RCS metabolic pathway and by activating GPX4 and SOD2 transcription. ALDH1A1-induced upregulation of SOD2 and GPX4, as well as ALDH1A1 itself, mitigated erlotinib-induced oxidative and carbonyl stress, and imparted resistance against the TKI. The elucidation of previously unrecognized metabolic mechanism underlying erlotinib resistance provides new insight into the biology of molecular targeted therapies and help to design improved pharmacological strategies to overcome the drug resistance.

## Abbreviations

ALDH1A1: Aldehyde dehydrogenase 1 family member A1
BSO: buthionine sulfoximine
CSC: cancer stem cell
CTC: circulating tumor cell
EGFR: epidermal growth factor receptor
EMT: epithelial-mesenchymal transition
GPX: glutathione peroxidase
GSH: glutathione
LC-QqQ-MS/MS: liquid chromatography coupled to triple-quadrupole tandem mass spectrometry
RCS: reactive carbonyl species
ROS: reactive oxygen species
SOD: superoxide dismutase
SRM: selective reaction monitoring
NAC: N-acetyl-L-cysteine
TKI: tyrosine kinase inhibitor
