# Aldehyde dehydrogenase 1A1 confers erlotinib resistance via facilitating the reactive oxygen species-reactive carbonyl species metabolic pathway in lung adenocarcinomas: Erratum

**DOI:** 10.7150/thno.84874

**Published:** 2023-04-15

**Authors:** Hui-Min Lei, Ke-Ren Zhang, Cong Hui Wang, Yang Wang, Guang-Lei Zhuang, Li-Ming Lu, Jian Zhang, Ying Shen, Hong-Zhuan Chen, Liang Zhu

**Affiliations:** 1 Department of Pharmacology and Chemical Biology, Shanghai Jiao Tong University School of Medicine, Shanghai, 200025, China.; 2 Shanghai Collaborative Innovation Center for Translational Medicine, Shanghai, 200025, China; 3 State Key Laboratory of Oncogenes and Related Genes, Ren Ji Hospital, School of Medicine, Shanghai Jiao Tong University, Shanghai, 200127, China.; 4 Central laboratory, Shanghai Chest Hospital, Shanghai Jiao Tong University, Shanghai, 200030, China; Shanghai Institute of Immunology, Shanghai Jiao Tong University School of Medicine, Shanghai, 200025, China.; 5 Department of Pathophysiology, Key Laboratory of Cell Differentiation and Apoptosis of National Ministry of Education, Shanghai Key Laboratory of Tumor Microenvironment and Inflammation, Shanghai Jiao-Tong University School of Medicine, Shanghai 200025, China.

Four transwell sub-pictures in Figure 6 (6B, siSOD2 Mock treatment on EV group and Mock treatment on ALDH1A1 group; 6C, siGPX4 Mock treatment on EV group; and 6H, NAC+siALDH1#4 treatment on HCC827 cells) were inadvertently misplaced. The correct version is shown below. Additionally, we would like to modify the legend text for Supplementary Figure S1(G) to read, “Expression of the ALDH1A1 protein was confirmed by western blot analysis which was performed concurrently with the western analysis shown in Figure 2A”.

The corrigendum does not affect any result or conclusion of the paper. The authors sincerely apologize for any inconvenience this may have caused.

## Figures and Tables

**Figure 6:**
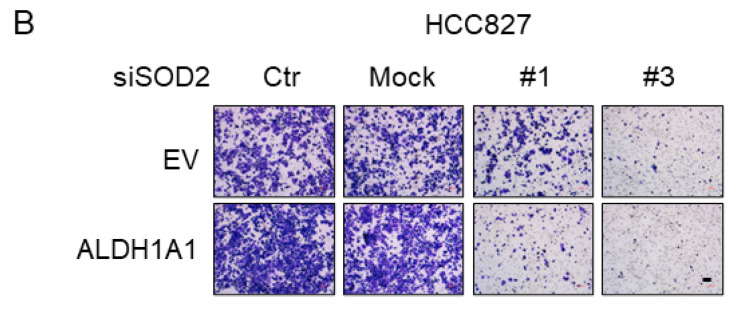

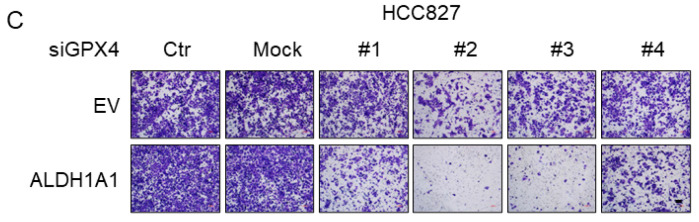

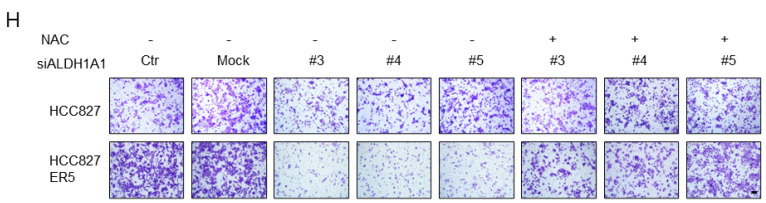
Corrected Figure 6 B, C, and H

