# Supplementary material for: Aldehyde dehydrogenase 1A1 confers erlotinib resistance via facilitating the reactive oxygen species-reactive carbonyl species metabolic pathway in lung adenocarcinomas
Source: Theranostics. 2019 Sep 23;9(24):7122–39. doi: 10.7150/thno.35729 (PMC6831290; doi:10.7150/thno.35729)
Supplement: Supplementary file 1 — Supplementary figures and tables. [file thnov09p7122s1.pdf]

Supplementary figures and figure legends

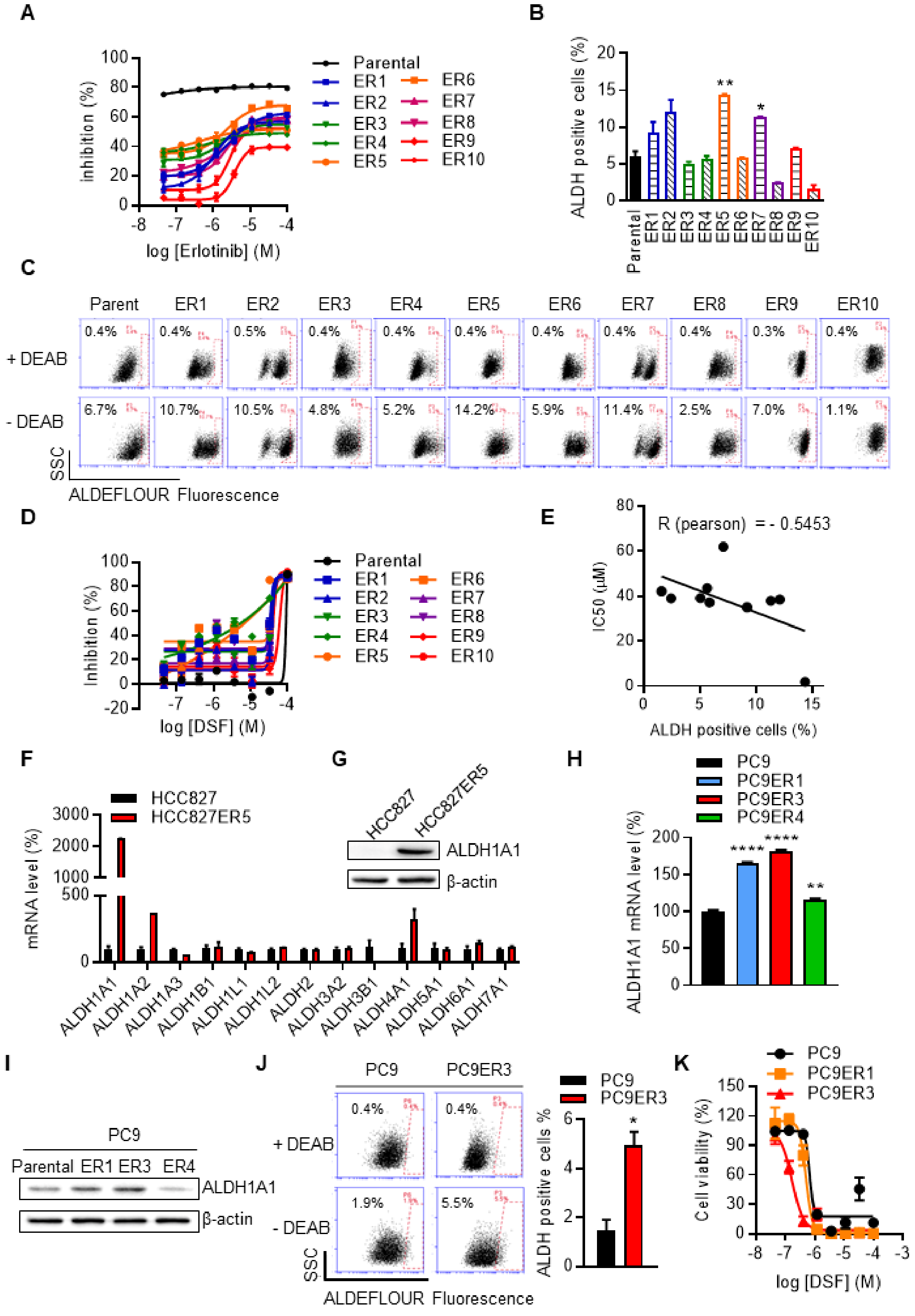

Supplementary Figure S1. ALDH1A1 dependence in erlotinib-resistant lung

adenocarcinoma cells.

(A) Inhibitory effect of erlotinib on cell viability in parental HCC827 cells and erlotinib-resistant variants (HCC827-ER1–ER10), as determined by performing CCK8 assays. The cells were exposed to erlotinib for 72 h. (B) Percentage of $ALDH1^+$ cells in parental HCC827 cells and erlotinib-resistant variants, as determined by performing ALDEFLUOR assays. (C) Representative flow cytometric data. N,N-diethylaminobenzaldehyde (DEAB), a selective ALDH1 inhibitor, was used as a control to define the ALDH activity. (D) Concentration-dependent inhibitory effect of DSF (a selective ALDH1 inhibitor) on the viability of parental HCC827 cells and erlotinib-resistant variants. The cells were exposed to DSF for 72 h. (E) Correlation between the percentage of $ALDH1^+$ HCC827 cells variants and the half-maximal inhibitory concentration (IC_{50}) of DSF for inhibiting these cells. The data shown were analyzed using Pearson’s correlation method. (F) Levels of mRNA of ALDH subtypes in HCC827 and HCC827-ER5 cells, as measured by RT-qPCR. (G) Expression of the ALDH1A1 protein was confirmed by western blot analysis. (H–J) The ALDH1A1 mRNA-expression levels (H), protein-expression levels (I), and activities (J) in parental PC9 cells and erlotinib-resistant variants. (K) Concentration-dependent effect of DSF on the viability of parental PC9 cells and erlotinib-resistant variants.

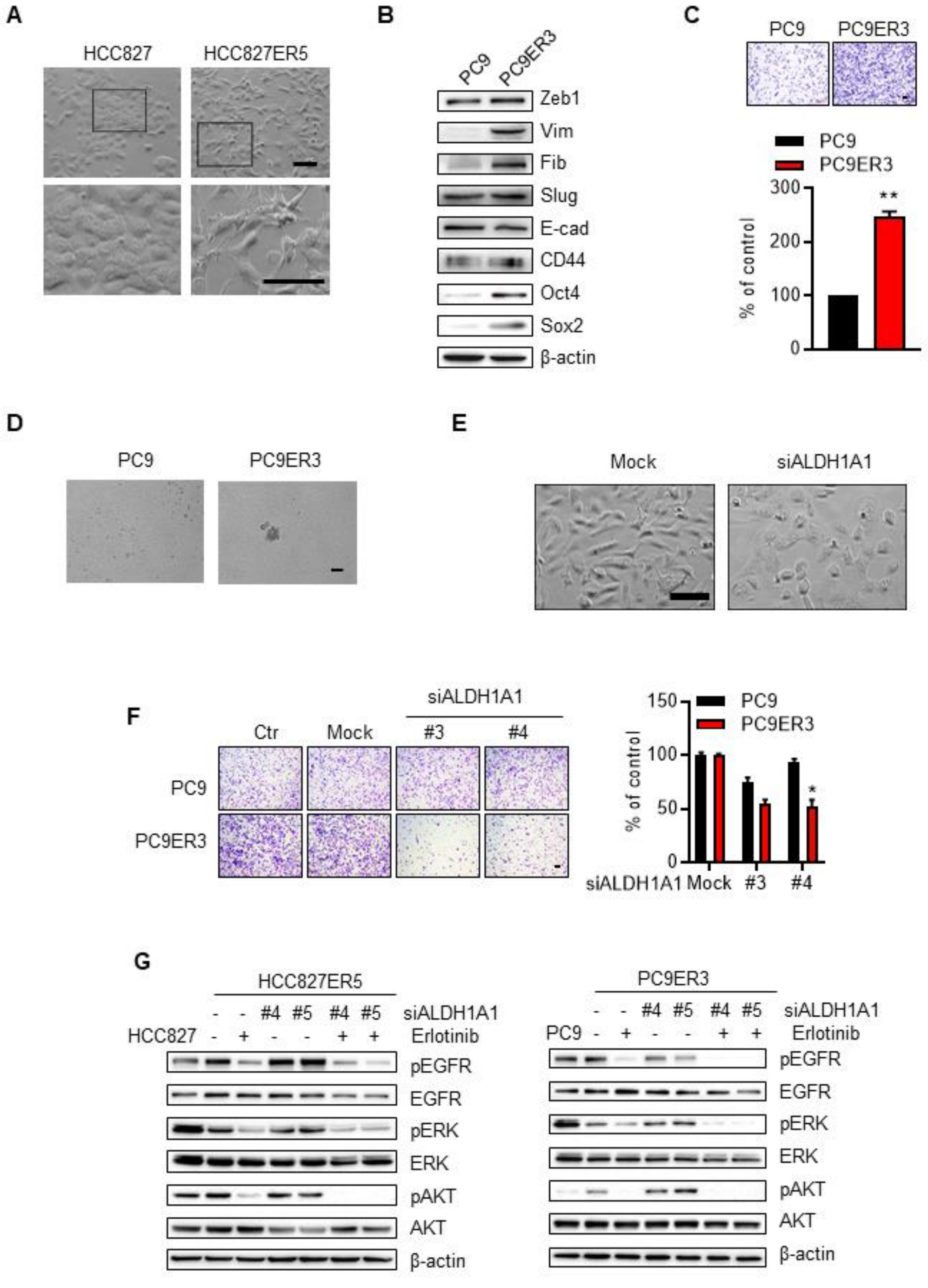

Supplementary Figure S2. Elevated EMT/CSC properties of the resistant cells depend on ALDH1A1.

(A) Representative morphological phase-contrast images of HCC827 and HCC827ER5 cells. Scale bar: 100 μm. (B) Western blot analysis of the mesenchymal markers Zeb1, vimentin, fibronectin, and Slug; the epithelial marker E-cadherin; and the cancer-stemness markers CD44,

Oct4, and Sox2 in PC9 and PC9ER3 cells. (C) Increased migration ability of PC9ER3 cells, as measured by performing transwell-migration assays. Scale bar: 100 µm. (D) Increased tumorsphere-formation ability of PC9ER3 cells compared with PC9 cells. Scale bar: 100 µm. (E) Representative morphological phase-contrast images of HCC827ER5 cells treated with siALDH1A1 for 48 h. (F) ALDH1A1 knockdown reversed the increased migration ability of PC9-ER3 cells. PC9-ER3 cells were transfected with 30 nM siRNA for 6 h, and transwell-migration assays were performed in fresh media lacking siRNA. Mock data of each corresponding cell line as control. (G) Western blot analysis of EGFR signaling. The cells were exposed to 1 µM erlotinib for 2 h, or transfected with 20 nM ALDH1A1 siRNA for 48 h, or the combination of erlotinib and siRNA-ALDH1A1.

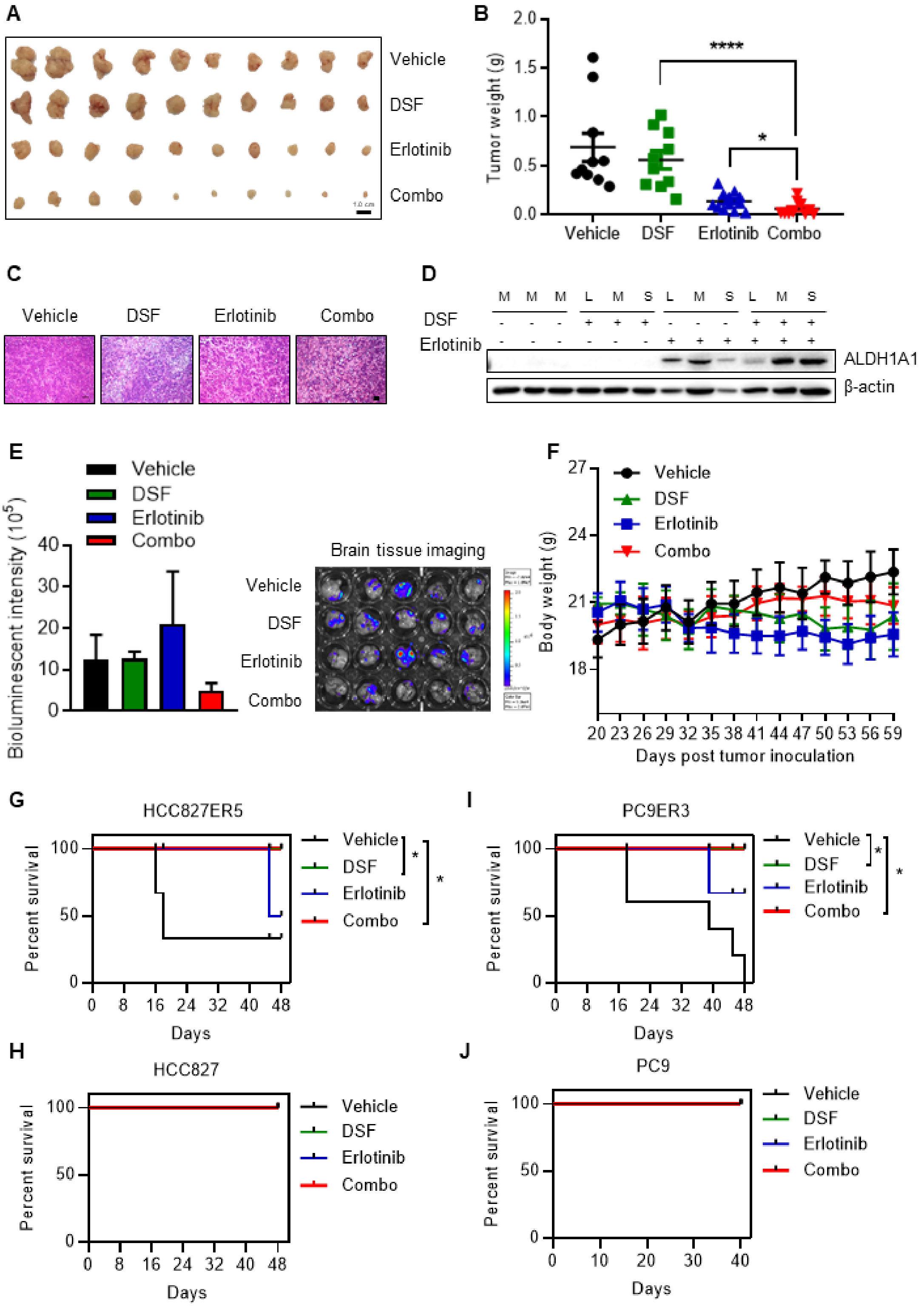

Supplementary Figure S3. **ALDH1 inhibition overcomes erlotinib resistance *in vivo*.**

(A–F) HCC827 CDX tumor-bearing mice were analyzed in terms of tumor imaging (A, scale bar: 1.0 cm), tumor weight (B), H&E staining (C), western blot analysis of ALDH1A1 protein

expression (D), bioluminescence imaging analysis of brain metastasis (E) and body weight change (F). (G-J) Mice transplanted with erlotinib-resistant cells died much earlier and ALDH1 inhibition substantially prolonged mice survival. Three million cells were subcutaneously injected into the flanks of mice, and the survival time of mice (n=5 per group) transplanted with HCC827ER5 (G), HCC827 (H), PC9ER3 (I), or PC9 (J) was recorded. The survival curves were analyzed by using GraphPad Prism software. All of mice transplanted with HCC827 or PC9 cells survived during the test time. Erlotinib, ALDH1 inhibitor DSF, or their combination had no effect on survival of mice transplanted with HCC827 (H) or PC9 (J) cells; DSF, or DSF combined with erlotinib, but not erlotinib prolonged survival of mice transplanted with HCC827ER5 (G) or PC9ER3 (I).

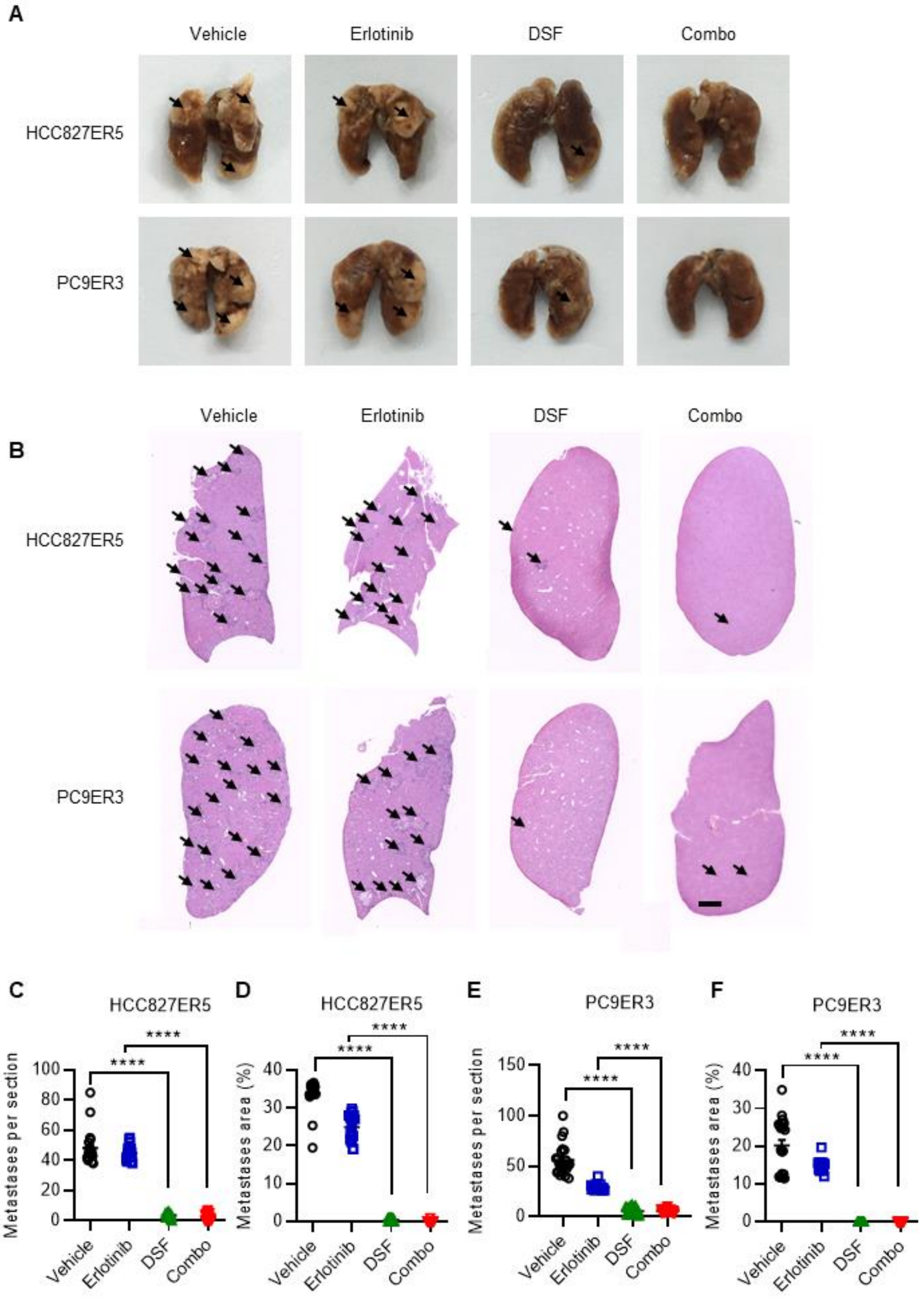

Supplementary Figure S4. ALDH1 inhibition suppresses metastasis of erlotinib-resistant cells *in vivo*.

(A) Suppression of lung dissemination of erlotinib-resistant cells by ALDH1 inhibition. Isolated representative lungs were from mice in Fig. 3G, I. The representative disseminated tumors are indicated by arrows. Photographs are 1× original magnification. (B-F) Suppression of liver metastasis of erlotinib-resistant cells by ALDH1 inhibition. Representative H&E staining of liver sections were from mice in Fig. 3G, I; Metastases are indicated by arrows; scale bar, 1 mm (B). Twenty sections of liver per group (4 sections per liver, 5 mice per group) were quantified for the number of liver metastasis per section (C, E) and the percentage of metastatic area per liver section (D, F). Data are presented as mean ± SEM.

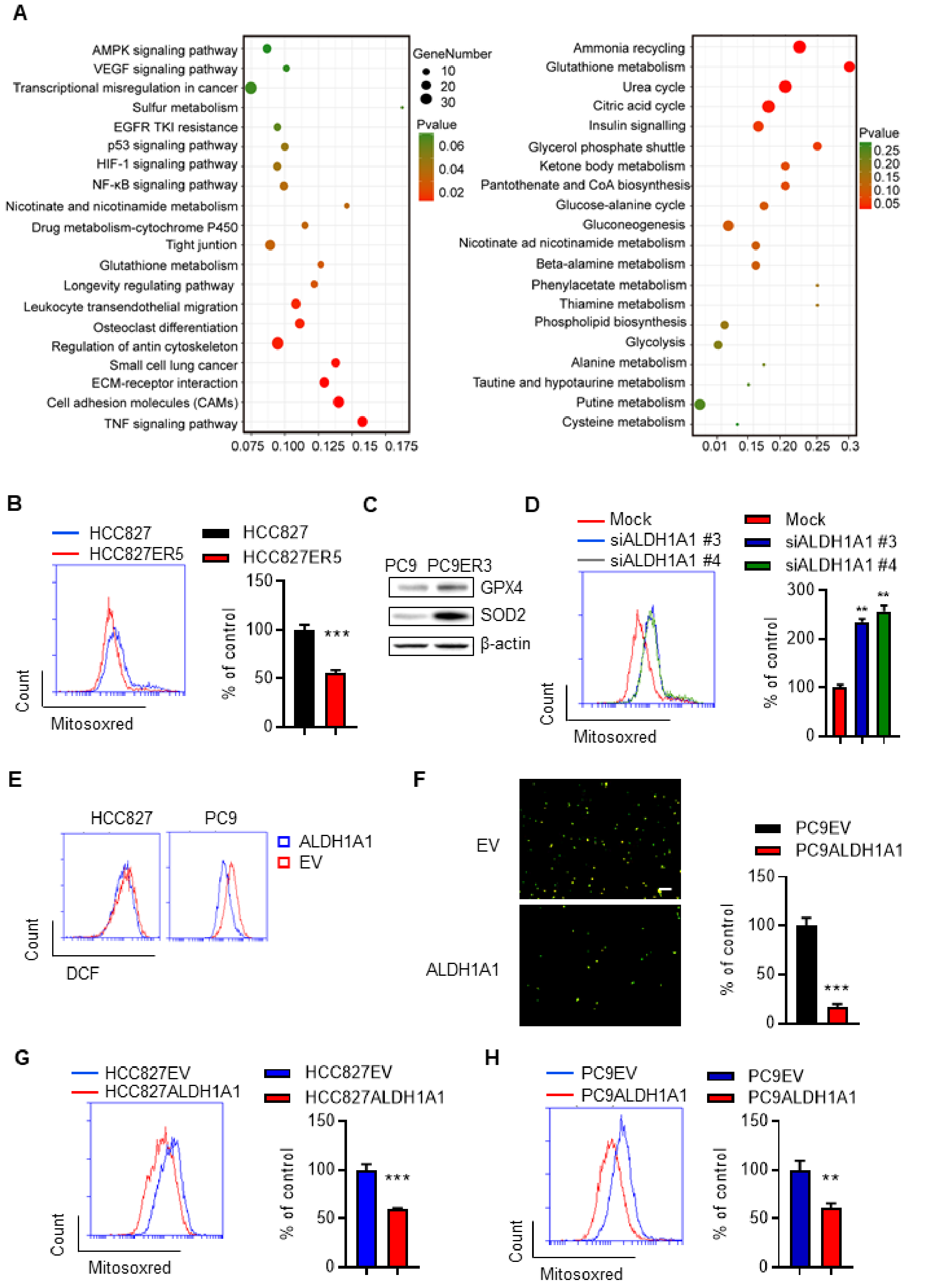

Supplementary Figure S5. **ALDH1A1-addicted erlotinib-resistant cells evolve an enhanced anti-ROS/RCS system.**

(A)Transcriptomic (left panel) and metabolomic (right panel) analysis based on RNA-Seq and

high-resolution mass spectrometry, respectively, of HCC827-ER5 versus parental cells. KEGG-pathway cluster analysis based on 1343 genes whose expression levels significantly changed. Untargeted metabolomics analyses were performed using LC-qTOF-MS/MS. The data was acquired and processed using MultiQuant software, version 2.0 (AB Sciex). Metabolites showing significantly different levels HCC827-ER5 cells compared with HCC827 cells were subjected to metabolic pathway-enrichment analysis using MetaboAnalyst 4.0 (http://www.metaboanalyst.ca/). (B) Mitochondrially generated ROS in HCC827 and HCC827ER5 cells were determined using the MitoSOX Red Mitochondrial Superoxide Indicator. (C) Western blot analysis of GPX4 and SOD2 expression in PC9 and PC9ER3 cells. (D) ALDH1A1 knockdown induced the accumulation of mitochondrially generated ROS. The cells were transfected with 30 nM siALDH1A1 or control siRNA for 48 h and stained with MitoSOX Red for flow cytometric analysis. (E) DOX-induced ALDH1A1 expression decreased intracellular ROS levels in PC9 and HCC827 cells assayed by DCFH-DA staining-based flow cytometry analysis. (F) DOX-induced ALDH1A1 overexpression decreased the intracellular levels of ROS. The cells were stained with DCFH-DA, imaged *in situ*, and analyzed using an IncuCyte living cell cytometer. (G-H) DOX-induced ALDH1A1 overexpression decreased mitochondrially generated ROS in HCC827 (G) and PC9 (H) cells assayed by MitoSOX Red-based flow cytometry analysis.

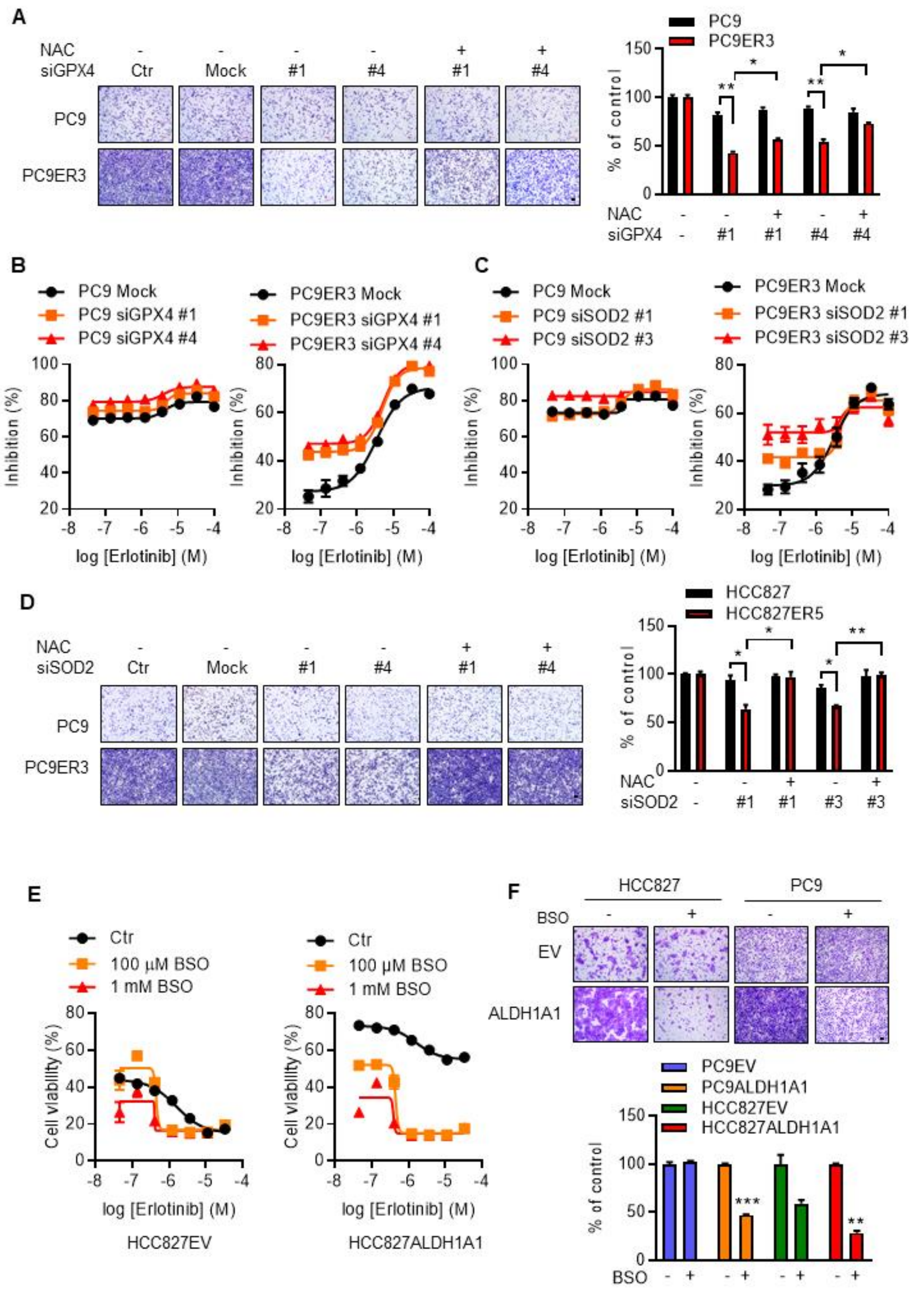

Supplementary Figure S6. **ALDH1A1-confered resistance to erlotinib depended on the ROS–RCS-metabolic pathway.**

(A and D) Knockdown of GPX4 (A) and SOD2 (D) selectively inhibited elevated migration in

PC9ER3 cells, and these effects were reversed by the ROS and RCS scavenger, NAC. The cells were transfected with siGPX4, siSOD2, or control siRNAs for 72 h. NAC (10 mM) was added 6 h before the point of the migration measurement. Mock data of each corresponding cell line as control. (B-C) Knockdown of GPX4 (B) or SOD2 (C) selectively re-sensitized PC9ER3 cells to erlotinib. The cells were transfected with siGPX4, siSOD2, or control siRNAs for 72 h. NAC (10 mM) was added 6 h before the point of the cell viability measurement. (E) DOX-induced ALDH1A1 expression decreased the inhibitory effect of erlotinib on HCC827 (right panel) cells compared with empty vector-transfected cells (left panel), as determined by performing CCK8 cell-viability assays, and this effect was abrogated by the GSH-synthesis inhibitor, BSO. The cells were exposed to erlotinib for 72 h. BSO was added 12 h before the point of the cell viability measurement. (F) DOX-induced ALDH1A1 expression increased the migration of HCC827 and PC9 cells, and these effects were reversed by a 12-h treatment with 100 μM BSO. BSO free data of each corresponding cell line as control.

Supplementary tables

Supplementary table S1. Oligonucleotides used in this study.

Oligonucleotides	
Real-time RCR	
ALDH1A1 forward primer	5'-GCTACTTTGTCCAGCCCACA-3'
ALDH1A1 reverse primer	5'-TCCCAGTTCTCTTCCATTTCC-3'
GPX4 forward primer	5'-GGACGACTGGCGCTGTGC-3'
GPX4 reverse primer	5'-TGGTGACGATGCACACGAAGC-3'
SOD2 forward primer	5'-CCTAACGGTGGTGGAGAACC-3'
SOD2 reverse primer	5'- CTGAGCCTTGGACACCAACA-3'
ALDH1L1 forward primer	5'-TGGTGGGTGTGTTCACTGTT-3'
ALDH1L1 reverse primer	5'-AATGAGGGTCCAGTTGATGG-3'
ALDH1L2 forward primer	5'-AGAAATGTGGTGGGCTTCAG-3'

ALDH1L2 reverse primer	5’-ATCAACATCCGCCAAAGAAG-3’
ALDH1B1 forward primer	5’-CAATGAATGGCAAGATGCAG-3’
ALDH1B1 reverse primer	5’-GACCTCATCCAAGTCCAAGG-3’
ALDH1A3 forward primer	5’-CGAGAGTGGGAAGAAGGAAG-3’
ALDH1A3 reverse primer	5’-AGGGCGTTGTAGCAGTTGAT-3’
ALDH1A2 forward primer	5’-TTAAGCCAGCAGAGCAAACA-3’
ALDH1A2 reverse primer	5’-GTCCAAGTCAGCATCAGCAA-3’
ALDH2 forward primer	5’-ATGGCAAGCCCTATGTCATC-3’
ALDH2 reverse primer	5’-TTGATCAGGTTGGCCACATA-3’
ALDH3B1 forward primer	5’-CGGTCTTCATCTGGAAGGAA-3’
ALDH3B1 reverse primer	5’-CATGACAATCTTGCCCACAC-3’
ALDH3A2 forward primer	5’-CGCTACATAGCCCCAACAGT-3’
ALDH3A2 reverse primer	5’-TTCCGTGATAAGCTCCCATC-3’
ALDH4A1 forward primer	5’-GTCCTTTGCCCGTATCAAGA-3’
ALDH4A1 reverse primer	5’-CTCCTGCACGACGTCCTTAT-3’
ALDH5A1 forward primer	5’-ACCAATTCTTGGTGCAAAGG-3’
ALDH5A1 reverse primer	5’-AGGCCCGAAAGTCTCTTCAT-3’
ALDH6A1 forward primer	5’-TCCAAGGTGAAATCCAGTCC-3’
ALDH6A1 reverse primer	5’-GGGTCTTCCCTTGTTCCAAT-3’
ALDH7A1 forward primer	5’-CAGTATGCGTGGCTGAAAGA-3’
ALDH7A1 reverse primer	5’-TTCCCCATCTCCAAAGACAC-3’
β-actin forward primer	5’-CGGGAAATCGTGCGTGAC-3’
β-actin reverse primer	5’-TGGAAGGTGGACAGCGAGG-3’
siRNA	
siALDH1A1 #3	CAAGAAATTTCCTGTCTTT
siALDH1A1 #4	GAGTGTTTACCAAAGACAT
siALDH1A1 #5	CATTTCTTCTCACATGGAT
siGPX4 #1	GGCAAGACCGAAGTAAACT
siGPX4 #2	TGGATGAAGATCCAACCCA

siGPX4 #3	AAGTTCCTCATCGACAAGA
siGPX4 #4	CCGATACGCTGAGTGTGGT
siSOD2 #1	AAAAGCTATTTGGAATGTA
siSOD2 #2	CAGCCTGCACTGAAGTTCA
siSOD2 #3	CTAATGATCCCAGCAAGAT

Supplementary table S2. IC50 of erlotinib on HCC827 cells and their erlotinib-resistant variants.

Cell variants	Parental	ER1	ER2	ER3	ER4	ER5	ER6	ER7	ER8	ER9	ER10
IC50 (μM)	0.026	2.41	1.08	1.82	8.50	3.47	2.32	2.56	2.62	3.76	2.72

The viability assay was performed by CCK8 analysis. The cells were exposed to erlotinib for 72 h.

Supplementary methods

Metastasis analysis

A total of 3×10^6 cells suspended in 100 μL PBS were subcutaneously inoculated into the left and right flanks of 5-week-old BALB/c nu/nu athymic mice. Metastasis of the subcutaneously inoculated tumors was analyzed by detecting the disseminated tumor foci in distant tissues. Tissues were removed for pathological analysis at the end point of the test or when the mouse was to die. After formalin-fixed 4-μm-thick sections were cut for hematoxylin and eosin (H&E) stain, the number and the area of disseminated tumor foci were counted and measured by using the NanoZoomer-S210 system (Hamamatsu, Japan). In bioluminescent imaging assay for metastasis, HCC827 cells transfected with luciferase (3×10^6) were subcutaneously inoculated into the left and right flanks of 5-week-old BALB/c nu/nu athymic mice. At the end of the test, the brains of the mice were isolated for detection of tumor metastasis basing on bioluminescent imaging analyzed by using the IVIS system (PerkinElmer, USA).

RNA-Seq analysis

Total RNA was extracted according to the manufacturer's instructions (Takara, 9767) from the indicated cell samples. RNA was subjected to RNA-Seq analysis on a BGISEQ-500 system by the Beijing Genomics Institute (BGI). FPKM was used to calculate the gene expression level. Differentially expressed gene screening was performed using the Noiseq method. Gene Ontology (GO) annotation was based on the GO database (http://www.geneontology.org/). The KEGG pathway database (http://www.genome.jp/kegg/) was used to perform the pathway enrichment analysis of differentially expressed genes.

Untargeted metabolomics

Sample preparation: Cells were seeded in 6-well plates (2×10^5/well) in triplicate and allowed to adhere overnight. Cell samples were extracted with 1 mL of MeOH: H_2O mixture (8:2, v/v) and centrifuged for 15 min at 20,000 × *g* and 4 °C to settle any particulate matter. Supernatants were taken separately for LC–MS/MS analysis. For UPLC/MS samples, three biological replicates were analyzed.

LCMS/MS analysis: The analysis was performed on a SHIMADZU Nexera UPLC System (consisting of a vacuum degasser, auto-sampler with thermostat, and binary pump) coupled to a TripleTOF™ 5600+ (AB Sciex). The separation of metabolites was carried out on a Waters ACQUITY UPLC BEH Amide column (2.1 mm × 100 mm, 1.7 µm). The mobile phase consisted of solvent A (0.1% aqueous ammonia and 20 mM ammonium carbonate in water) and solvent B (acetonitrile). The flow rate was set at 0.4 mL/min. The total elution time was 15 min and the elution condition was set as following: 0–2 min, 5% A; 2–9 min, 5%→55% A; 9–11 min, 55% A; including a 4 min equilibration time at 5% A. The metabolites were ionized using the electrospray ionization interface operating in negative ion mode and positive ion mode. IonSpray voltage was set at −4500 V (ESI-) and 5500 V (ESI+), curtain gas was kept at 35 psi, ion source temperature was 500 °C (ESI-) and 550 °C (ESI+), and nebulizing gas and drying gas were 55 psi. The collision energies were optimized with respect to the analyte between 15 eV and 45 eV to maximize the analyte response. Data was acquired and processed using MultiQuant software version 2.0 (AB Sciex).
